# The MukB-topoisomerase IV interaction mutually suppresses their catalytic activities

**DOI:** 10.1093/nar/gkab1027

**Published:** 2021-11-08

**Authors:** Rupesh Kumar, Soon Bahng, Kenneth J Marians

**Affiliations:** Molecular Biology Program, Memorial Sloan Kettering Cancer Center, 1275 York Avenue, New York, NY 10065, USA; Molecular Biology Program, Memorial Sloan Kettering Cancer Center, 1275 York Avenue, New York, NY 10065, USA; Molecular Biology Program, Memorial Sloan Kettering Cancer Center, 1275 York Avenue, New York, NY 10065, USA

## Abstract

The bacterial condensin MukB and the cellular chromosomal decatenase, topoisomerase IV interact and this interaction is required for proper condensation and topological ordering of the chromosome. Here, we show that Topo IV stimulates MukB DNA condensation by stabilizing loops in DNA: MukB alone can condense nicked plasmid DNA into a protein–DNA complex that has greater electrophoretic mobility than that of the DNA alone, but both MukB and Topo IV are required for a similar condensation of a linear DNA representing long stretches of the chromosome. Remarkably, we show that rather than MukB stimulating the decatenase activity of Topo IV, as has been argued previously, in stoichiometric complexes of the two enzymes each inhibits the activity of the other: the ParC subunit of Topo IV inhibits the MukF-stimulated ATPase activity of MukB and MukB inhibits both DNA crossover trapping and DNA cleavage by Topo IV. These observations suggest that when in complex on the DNA, Topo IV inhibits the motor function of MukB and the two proteins provide a stable scaffold for chromosomal DNA condensation.

## INTRODUCTION

Condensation and topology of the bacterial chromosome is managed mainly by the action of three distinct types of proteins: condensins, topoisomerases, and nucleoid associated proteins (1–3). In *Escherichia coli*, the condensin, MukB, and the primary chromosome decatenating enzyme, topoisomerase IV (Topo IV), interact (4,5) and this interaction was shown to be required for proper chromosome condensation (6).

**Figure 1.**
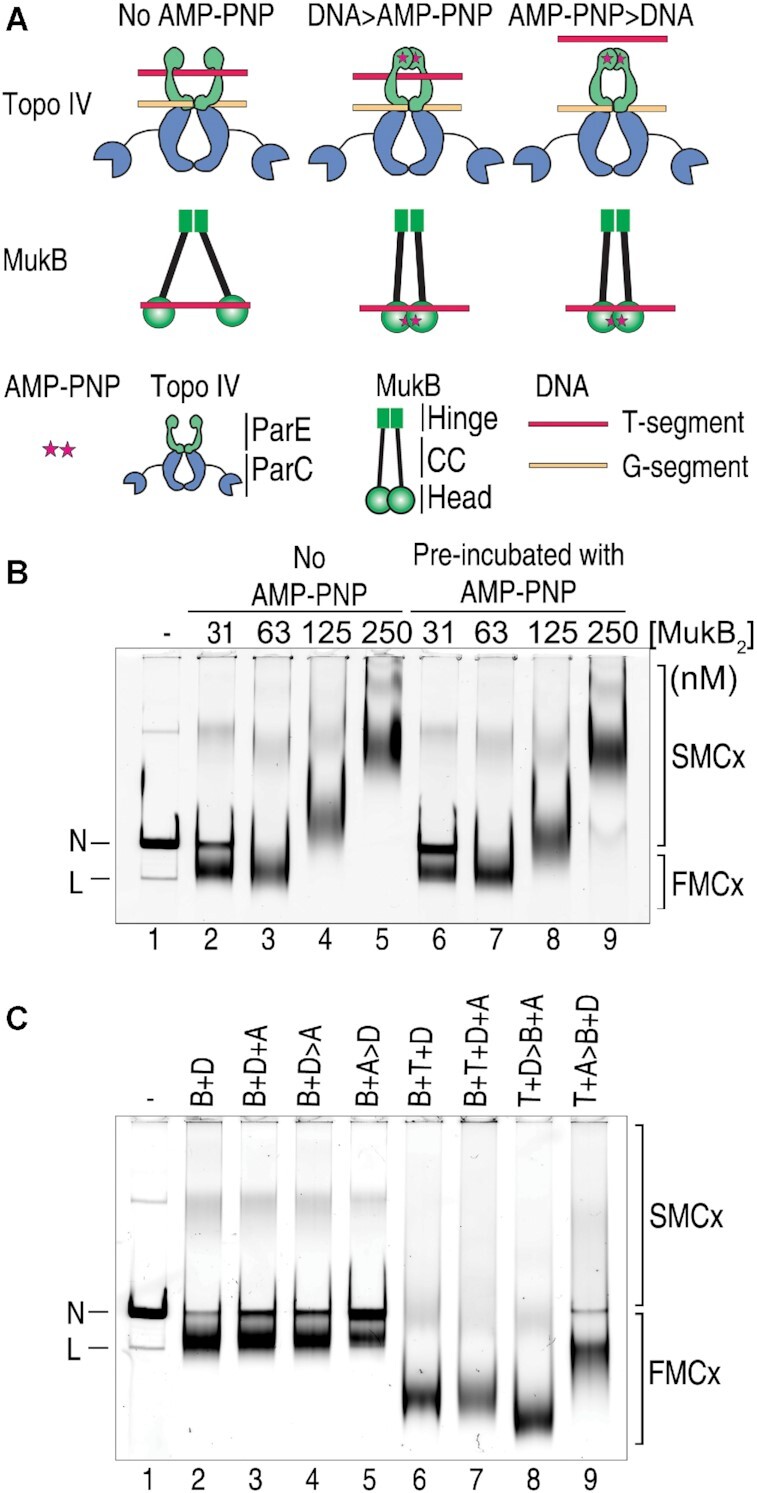
Effect of AMP-PNP on DNA condensation by MukB and Topo IV. (**A**) ATP-operated gates of Topo IV and MukB. Topo IV can bind two segments of DNA, the G segment (yellow), which binds across the ParC dimer at the catalytic tyrosines, and the T segment (red), that is captured by the dimerization of ParE monomers, forming the N gate, in the presence of ATP. MukB monomers dimerize at the hinge region, which is separated from the ATPase head domains by long coiled coils. The MukB head regions can dimerize in the presence of ATP and DNA can bind along the top (as drawn) of the head domains. DNA > AMP⋅PNP, the enzymes are exposed to DNA before AMP⋅PNP is added. AMP⋅PNP > DNA, AMP⋅PNP is added first to close either the Topo IV N gate or the MukB head domains followed by DNA. CC, coiled coil. (**B**) AMP⋅PNP does not inhibit MukB condensation of nicked plasmid DNA. MukB was pre-incubated either with or without 5 mM Mg-AMP⋅PNP for 5 min, added at the indicated concentrations to DNA condensation reaction mixtures containing singly-nicked plasmid DNA, incubated for an additional 5 min, and then analyzed as described under Materials and Methods. N, nicked plasmid DNA; L, linear plasmid DNA; SMCx, slow-moving complex; FMCx, fast-moving complex. (**C**) Closing of the ATP-dependent N gate of Topo IV before the enzyme is exposed to DNA prevents the generation of the hyper-condensed protein–DNA complex. Reaction mixtures containing 31.3 nM MukB, 62.5 nM Topo IV, and 5 mM Mg-AMP⋅PNP were analyzed for DNA condensation as described above and under Materials and Methods. Incubations were in two steps, denoted by the ‘greater than’ symbol. Letters at the top of each lane describe the order of addition of the reagents. B, MukB; D, nicked plasmid DNA; T, Topo IV; and A, AMP⋅PNP. For example: B + D > A indicates that MukB and DNA were incubated first for 5 min followed by the addition of AMP⋅PNP and an additional 5 min incubation before loading on the gel, whereas T + D > B + A indicates that Topo IV and DNA were incubated together first followed by the addition of MukB and AMP⋅PNP in the second incubation.

MukB is a Structural Maintenance of Chromosomes (SMC)-like protein comprised of two globular regions at the N- and C-terminus with a long α-helical section between them that is itself separated by a hinge region in the middle (7). The monomeric protomer folds at the hinge region with the globular regions associating to form the ATPase head region separated from the hinge by a long coiled-coil region of the α helices. The protein itself is dimeric, associating at the hinge regions of the protomers (8–10). The two head regions in the dimeric protein can associate by shared binding of ATP (Figure 1A) (11). In a fully extended structure, the hinge and head regions can be separated by 50 nm. The complete condensin is a tri-partite structure of a dimer of MukB, a dimer of the kleisin, MukF, and a dimer of the KITE (kleisin interacting winged-helix tandem elements) subunit, MukE (1,8,12). Cells carrying mutations in any of these components manifest with decondensed chromosomes, chromosome segregation defects, increased anucleate cell production, and are temperature sensitive (7,13,14).

Topo IV is a type II topoisomerase that is required for the terminal stages of DNA replication to unlink the replicating sister chromosomes (15–17). It is a heterotetramer composed of dimers of the breakage and reunion subunit ParC and the ATPase subunit ParE (18,19). Cells carrying mutations in either *parC* or *parE* are temperature-sensitive and display a chromosome-segregation *par* (partition) phenotype where an unsegregated mass of chromosomes in the center of the cell continues to replicate (15,20). Like all type II topoisomerases, Topo IV alters topology by capturing a segment of DNA (the T or transport segment) via the closing of the ParE subunits in the presence of ATP (forming the N gate) (Figure 1A) and passing it through a double-stranded break (the DNA gate) in another DNA segment (the G or gate segment) bound to the ParC subunits, which is where the active site tyrosines are located. The cleaved DNA in the DNA gate is then resealed and the T segment leaves the enzyme through the C or exit gate, which is also formed by the ParC subunits (21). If the T and G segments of DNA are derived from the same DNA molecule, the enzyme can relax both positive and negative supercoils. If the two segments are derived from two different DNA molecules, the enzyme can catenate and decatenate the DNAs (22). In addition to its catalytic role in managing DNA topology, we have shown that Topo IV also plays a role in chromosome organization (6,23)

Understanding how SMC proteins modulate DNA structure has been an enduring effort for many laboratories over the past two decades. SMC protein complexes have been shown to modulate DNA conformation by an ATP-dependent process called loop extrusion whereby the protein complex generates and expands loops of DNA (24–26), a reaction thought to be able to account for how these proteins organize chromosomes. It has also been demonstrated that SMC proteins can translocate on double-stranded DNA (27–29). Although such reactions have not yet been demonstrated for MukBEF, it has been shown that the condensin organizes the *E. coli* chromosome into an axial core of the protein from which loops of DNA extrude (30).

Using agarose gel electrophoretic assays, we demonstrated that in the absence of ATP, MukB alone could re-organize topologically unconstrained plasmid DNA into protein-sequestered negative supercoils and topologically constrained DNA loops (23), generating protein–DNA complexes that had a greater electrophoretic mobility than the nicked plasmid DNA. MukB-condensed plasmid DNA visualized by atomic force microscopy resembled the organization of chromosomal DNA in vivo (23) The addition of Topo IV further condensed these protein–DNA complexes, in a reaction requiring the interaction between ParC and MukB, to generate an even faster-moving protein–DNA complex, whereas the addition of MukE and MukF had little effect (6).

Here, we report that of the two ATP-operated gates present in a Topo IV-MukB-DNA complex, only the Topo IV N gate is required to generate the DNA condensation observed by gel electrophoresis. In addition to stimulating DNA condensation by interaction with MukB molecules, Topo IV generates a preferred substrate for MukB-DNA condensation by stabilizing a DNA crossover. Furthermore, the interaction between Topo IV and MukB results in mutual inhibition of individual activities of the proteins: The ParC subunit of Topo IV inhibits the MukF-stimulated ATPase activity of MukB in vitro and MukB inhibits capture of the T segment in vitro and the DNA cleavage activity of Topo IV in vivo. These observations suggest that the MukB-Topo IV complex forms a stable scaffold for chromosome organization in the cell.

## MATERIALS AND METHODS

### Proteins and DNA

MukB (4), MukE, MukF (31), ParC and ParE (32) were purified as described previously. Cesium chloride gradient-purified supercoiled pCG09 DNA (33) was either nicked or linearized using Nb.BbVCI and ScaI, respectively, as described (31).

### DNA condensation assays

As described previously (34). Briefly, nicked (Nb.BbVCI) or linear (ScaI) ∼10 kb-long pCG09 plasmid was used as a substrate for DNA condensation by MukB in the presence and absence of either ParC or Topo IV. Reaction mixtures containing 50 mM HEPES–KOH (pH 7.5), 20 mM KCl, 10 mM 1,4-dithiothreitol (DTT), 0.5 mM Mg(OAc)_2_ and the indicated concentrations of proteins were incubated for 5 min at 37°C. DNA–protein complexes were resolved by electrophoresis through 0.8% agarose gels at 4°C for 18 h at 1.8 V/cm using 50 mM Tris–HCl (pH 7.8), 40 mM NaOAc, 1 mM EDTA, and 1.5 mM Mg(OAc)_2_ as the electrophoresis buffer. In assays to test the effect of AMP⋅PNP, either Topo IV or MukB were preincubated in the presence of 5 mM AMP⋅PNP and an additional 5 mM Mg(OAc)_2_ for 5 min before the addition of DNA. Gels were stained with SYBR Gold (Thermo Fisher Scientific) and imaged using a Typhoon FLA 9500 fluorescent scanner from GE Healthcare.

For southwestern analysis, the protein–DNA complexes were transferred electrophoretically at 30 V for 18 h to PVDF membranes in transfer buffer containing 25 mM Tris, 192 mM glycine and 20% methanol at 4°C. The blot was fixed with 0.7% acetic acid in water, followed by neutralization with phosphate buffered saline containing 0.05% Tween 20. The blot was probed with either affinity-purified ParC or MukB antibody and developed using Amersham ECL western blotting reagents. The blots were imaged using a Biorad Chemidoc system and Multi Gauge V2.3 software was used for densitometric quantification.

### Trapping of right-handed crossovers by topo IV

Nicked DNA resealing, as described previously (23), was used to monitor topological changes induced in the DNA upon binding of Topo IV and MukB. In this assay, 62.5 nM of catalytically inactive Topo IV (reconstituted by mixing Y120F ParC with ParE) was incubated with nicked DNA in the presence of varying concentrations of MukB for 5 min at 37°C in the buffer used for the DNA condensation assay, which was supplemented with 26 μM NAD. The nick in the DNA was sealed by the addition of *E. coli* DNA ligase (New England Biolabs, 0.4 unit) followed by incubation for 5 min at the same temperature. The reactions were stopped by the addition of NaCl to 300 mM and EDTA to 25 mM. Subsequently, SDS and proteinase K were added to a final concentration of 0.2% and 0.2 mg/ml, respectively, and the reactions were incubated for 30 min at 37°C. Resulting topoisomers were resolved by electrophoresis through 0.8% agarose gels at 1.8 V/cm for 18 h using 50 mM Tris–HCl (pH 7.8), 40 mM NaOAc, 1 mM EDTA as the electrophoresis buffer. Gels were stained with SYBR Gold and imaged using a Typhoon FLA 9500 scanner. Note that the difference between the temperatures of the reactions (37°C) and the gel electrophoresis (room temperature) results, because of unwinding of the double helix as a function of increased temperature, in a change of roughly four positive supercoils in the topology of the DNA. This effect is equal for the control lane and the reaction lanes of the gel in Figure 4 and thus does not affect our conclusions.

### ATPase assay

As per (31). Reaction mixtures (10 μl) containing 50 mM HEPES–KOH (pH 7.5), 20 mM KCl, 10 mM DTT, 2 mM Mg(OAC)_2_, 2 mM ATP, 2 μCi [γ-^32^P]ATP, 100 μg/ml BSA, and the indicated concentrations of MukB, MukF, and ParC were incubated for 10 min at 37°C. Reactions were terminated by the addition of EDTA to 100 mM and an aliquot (1 μl) chromatographed on PEI cellulose plates (Millipore) using 0.5 M LiCl, 1 M HCOOH as the developing buffer. Plates were dried, exposed to a phosphorimager screen, and the screen was scanned in a GE Typhoon FLA 9500 Scanner. The fraction of ATP hydrolyzed was quantified using Image Gauge software (Fuji).

### MukB-DNA Pull-down assay

A tailed, nicked circular DNA was prepared as described (35) using the oligonucleotide primer 5′-Bio-Bio-AGAGAGTTACCGATAGAGTTAGCCTGCAATGATGTCAATAACCTGTTTAGCTATATTTTCATTTGGGG-3′ where the last 34 nt anneal to M13mp18 ssDNA. This DNA was digested with BglII to yield a linear DNA with a 5′-biotinylated ss DNA flap with 362 bp upstream and 6887 bp downstream of the flap. This DNA was conjugated to M280 magnetic beads (Dynabeads) as described by the manufacturer and stored in 10 mM Tris–HCl, pH 7.5, 1 mM EDTA, 0.05% NP-40. MukB was bound to the DNA in a reaction mixture containing 50 mM HEPES–KOH, pH 7.5, 20 mM KCl, 10 mM DTT, 7.5% glycerol, 0.5 mM Mg(OAc)_2_, 0.05% NP-40, 1 nM DNA conjugated to beads, and 200 nM MukB for 30 min at room temperature on a rotator. The beads were then pulled down on a magnet, the supernatant removed, and the beads resuspended in reaction buffer. MukB-DNA pull-down assays (20 μl) contained 150 nM MukB and were performed in the same reaction buffer in two steps: 1) either 600 nM MukEF, 600 nM ParC, or no additional protein was added and the reaction mixtures were incubated for 5 min at 37°C, then 2) either the protein not added in the first step or no additional protein was subsequently added and the incubation continued for an additional 5 min at 37°C. The MukB-DNA beads were then pulled down on a magnet and the supernatant removed. Proteins bound to the beads and present in the supernatant were then visualized by SDS-PAGE. Images were quantified using Fuji Image Gauge software.

### Norfloxacin survival and DNA damage assays

#### Generation of Strains

*gyrA^nalR^* strains of *E. coli* BW30270 containing a Ser83 to Leu substitution in *gyrA* were generated by P1 phage transduction with a lysate grown on E. coli C600 (*gyrAS83L zei‐723::Tn10)* (36). Deletion of *mukB* was carried out similarly using a P1 lysate grown on AZ5372 (*F^–^*, *trpC9941*, *ΔmukB::Kan*) (7). Norfloxacin-resistant colonies were selected on LB agar plates containing 50 ng/ml of norfloxacin and *mukB* deleted strains were selected on kanamycin (50 μg/ml) containing LB agar plates grown at room temperature.

#### Norfloxacin treatment, cell survival and pulsed field gel electrophoresis

Cells were grown at 250 rpm and 25°C until mid log phase in LB medium before the addition of varying concentrations of norfloxacin. Cultures were then incubated on the shaker for an additional 30 min, followed by harvesting of the cells. Cells were washed three times with fresh LB medium and dilutions were plated on LB agar plates without drug at 25°C to determine colony forming units. For pulsed field gel electrophoresis, washed cells were resuspended at 2.0 O.D._600_/ml, mixed with an equal volume of molten 1% agarose (∼50°C) in LB medium, and poured in a mold to prepare blocks. The solidified blocks were transferred to 1.5 ml tubes containing 50 mM Tris–HCl (pH 8.0), 10 mM EDTA (pH 8.0), 0.5% SDS and 0.5 mg/ml proteinase K, and incubated for 24 h at 37°C. The blocks were placed into similar-sized wells of an 0.8% agarose gel prepared in 25 mM Tris-borate, pH 8.3, 0.5 mM EDTA and sealed inside the wells by addition of 0.8% molten agarose. DNA fragments were resolved using a Biorad (CHEF-DR III) pulsed field gel electrophoresis system at constant 6 V/cm (120° angle, initial and final switch times 10 s and 60 s, respectively). Gels were visualized and densitometric quantifications performed as described above.

#### Fractionation of DNA bound and unbound topoisomerase IV

Cells were grown in LB medium to mid-log phase at 250 rpm and 25°C, harvested, washed with fresh LB medium and resuspended in a lysis buffer containing 100 mM Tris–HCl (pH 8.0), 50 mM NaCl, 10% (v/v) glycerol, 5 mM DTT, 20 mM EDTA and 0.02% lysozyme and incubated at 4°C for 30 min. Brij-58 was added to 0.1% and the incubation continued for 5 min. The cell suspension was then centrifuged at 100 000 × g for 60 min at 4°C. The supernatant was taken as the DNA-unbound fraction, whereas the pellet was taken as the DNA bound fraction. The pellet containing DNA and the proteins bound to it, was resuspended in the lysis buffer (without lysozyme) by sonication. Multiple dilutions of these fractions were mixed with SDS sample buffer containing 2-mercaptoethanol, heated for 10 min at 75°C, and resolved by electrophoresis through 3–12% Bis–Tris SDS polyacrylamide gels. Proteins were transferred to PVDF membrane, probed with ParC antibodies, and the blots developed as described above. The acetic acid step was omitted in this experiment.

#### MukB-ParC binding assay

Affinity-purified MukB polyclonal antibodies were bound to Dynabeads Protein A (Thermo) in PBS containing 0.02% Tween 20 (PBST), unbound antibodies were removed, and the beads washed in PBST. MukB was then bound to the beads for 1 h in PBST, unbound protein was removed, the beads washed, and resuspended in PBST. The amount of MukB immobilized on beads was determined by heating different volumes of bead suspension at 95°C for 5 min in 1× SDS sample buffer, followed by SDS-PAGE and comparison to known amounts of MukB on the same gel. Varying concentrations of either wild-type or R705E,R729A ParC were bound to a fixed concentration (125 nM) of MukB-beads in a 50 μl reaction volume containing 50 mM HEPES KOH, pH 7.5, 50 mM KCl, 0.5 mM Mg(OAc)_2_, 7.5% glycerol, and 0.05% IGEPAL for 15 min at 1250 rpm and 37°C in a thermomixer (Eppendorf). Unbound ParC was removed and the beads were washed with 1 ml, followed by 0.1 ml of reaction buffer. The proteins released from the beads were resolved on SDS polyacrylamide gels, visualized, and analyzed as described above. Band intensities were determined and quantified using Fiji (37). Binding data were fit to the Hill equation.

#### Purification of GyrA S83L

Three liters of BL21 DE3 (pET11c-GyrAS83L) was grown at 37°C in LB-medium to an O.D._600_ = 0.5. The temperature was then reduced to 16°C and protein expression induced by the addition of IPTG to 0.4 mM followed by continued incubation at 16 h. Cells were harvested, resuspended in Buffer A (50 mM Tris HCl, pH 7.4, 10% (v/v) Glycerol, 10 mM DTT and 1 mM EDTA) with 150 mM NaCl and lysed by the addition of EDTA (20 mM) and lysozyme (0.02%) followed by incubation for 20 min on ice. All subsequent steps were at 4°C. The lysate was centrifuged at 100 000 × g for 1 h and the supernatant recovered. Nucleic acids were precipitated the addition of Polymin P to 0.07% followed by centrifugation at 37 000 × g for 10 min. Protein in the supernatant was precipitated with ammonium sulfate (50% saturation) and recovered by centrifugation at 37 000 × g for 30 min. The pellet was resuspended in the buffer A + 25 mM NaCl, dialyzed against the same buffer, and loaded on a DEAE cellulose column (25 ml) equilibrated with Buffer A + 25 mM NaCl. After washing with two column volumes of the same buffer, protein was eluted using a 10 column volume linear gradient of 25–450 mM NaCl in buffer A. Fractions containing GyrA were identified by SDS-PAGE, pooled, dialyzed against buffer A + 50 mM NaCl, and loaded on a heparin agarose column (30 ml) equilibrated in buffer A + 50 mM NaCl. The column was washed with 2 column volumes of the same buffer A and protein eluted with a 10 column volume linear gradient of 50–600 mM NaCl in buffer A. Peak fractions containing GyrA were pooled, the protein precipitated with ammonium sulfate (55% saturation), collected by centrifugation, resuspended in 1.5 ml of buffer A + 500 mM NaCl, and gel filtered through a Superdex 200 (130 ml bed volume) column. Peak fractions were pooled, dialyzed against storage buffer (50 mM Tris–HCl, pH 7.4, 150 mM NaCl, 40% glycerol, 1 mM EDTA and 5 mM DTT), aliquoted, and stored at –80°C.

#### DNA gyrase supercoiling assay

DNA gyrase was reconstituted by mixing either wild type or S83L GyrA with a 25% excess of GyrB and incubating on ice for 30 min. Supercoiling reactions (20 μl) containing 50 mM HEPES–KOH, pH 7.5, 20 mM KCl, 10 mM DTT, 8 Mg(OAc)_2_, 0.1 mg/ml BSA, 5 μg/ml tRNA, 2 mM ATP, 0.34 nM pCG09, and the indicated concentrations of DNA gyrase and norfloxacin were incubated for 10 min at 37°C. The reactions were then terminated, processed, and analyzed as above for the nick resealing assays.

All assays shown in this report were repeated at least three times.

## RESULTS

### DNA condensation by MukB and topo IV requires closing of the topo IV N gate

As described in the Introduction, complexes of MukB and Topo IV on the DNA possess two ATP-operated gates: the N gate of Topo IV, which traps the T segment of DNA during its modulation of DNA topology and the ATPase heads of the MukB dimer that can close upon binding ATP (Figure 1A). The closing of SMC protein head domains is thought to divide the protein into two compartments that can bind DNA (38,39). To better understand the nature of the DNA condensation that we observe in the presence of MukB and Topo IV, we asked whether closing of either ATP-operated gate was required.

Condensation of a singly-nicked 11-kb plasmid DNA by MukB was compared in the absence or presence of pre-incubation of the protein with 5 mM AMP⋅PNP (Figure 1B and Supplementary Figure S1A), a non-hydrolyzable ATP analog that can dimerize the ParE subunits of Topo IV (40) and the ATPase heads of the MukB dimer (11). DNA condensation is assessed by electrophoresis of the protein–DNA complexes through agarose gels (23). The ATP analog inhibited MukB DNA binding slightly at low concentrations of MukB (Figure 1B and Supplementary Figure S1A, compare lanes 2 and 6), but did not prevent condensation of the DNA (Figure 1B and Supplementary Figure S1A, compare lanes 3 and 7). Increasing concentrations of MukB result in the binding of greater amounts of the protein to the DNA, resulting in the expected decrease in the mobility of the protein–DNA complex (slow-moving complex(es), SMCx in the figures) compared to that of the DNA alone (Figure 1B and Supplementary Figure S1A, lanes 4, 5, 8 and 9). These protein–DNA complexes contain topologically isolated DNA loops that can be supercoiled by DNA gyrase (23).

The addition of Topo IV to the MukB–DNA complexes described above results in the formation of protein–DNA complexes that migrate even faster through agarose gels than the MukB-DNA complexes (which we termed a hyper-condensed protein–DNA complex, fast-moving complex(es), FMCx in the figures) (6). We compared the role of closing of the ATP-operated gates in these complexes by order-of-addition experiments (Figure 1C and Supplementary Figure S1B). As shown in Figure 1B, pre-incubating AMP⋅PNP with MukB inhibited binding of the protein to the DNA to some extent (Figure 1B and Supplementary Figure S1A, compare lanes 2 and 6). Addition of AMP⋅PNP to the incubation of MukB and Topo IV in the presence of DNA had little effect on the formation of the highly condensed DNA (Figure 1C and Supplementary Figure S1B, compare lanes 6 and 7), as did addition of Topo IV to DNA followed by the addition of MukB pre-incubated with AMP⋅PNP (Figure 1C and Supplementary Figure S1B, compare lanes 6 and 8). In fact, this order of addition yielded a complex with the greatest mobility (Figure 1C and Supplementary Figure S1B, compare lane 8 with lanes 6, 7, and 9). On the other hand, pre-incubation of Topo IV with AMP⋅PNP followed by the addition of MukB and DNA yielded condensed protein–DNA complexes that migrated significantly slower than the Topo IV–MukB–DNA complex (Figure 1C and Supplementary Figure S1B, compare lanes 6 and 9) and only slightly faster than the MukB-DNA complex (Figure 1C and Supplementary Figure S1B, compare lanes 2 and 9). We conclude that closing of the N gate of Topo IV before the protein is exposed to DNA prevents the formation of the hyper-condensed DNA, whereas closing of the MukB ATP heads has no effect.

These observations indicated that Topo IV was increasing DNA condensation by trapping a T segment of DNA, creating a stabilized DNA crossover with a G segment, suggesting that Topo IV together with MukB might be able to condense even linear DNA by virtue of Topo IV creating topologically closed loops. This proved to be the case.

### MukB and Topo IV can condense linear DNA

**Figure 2.**
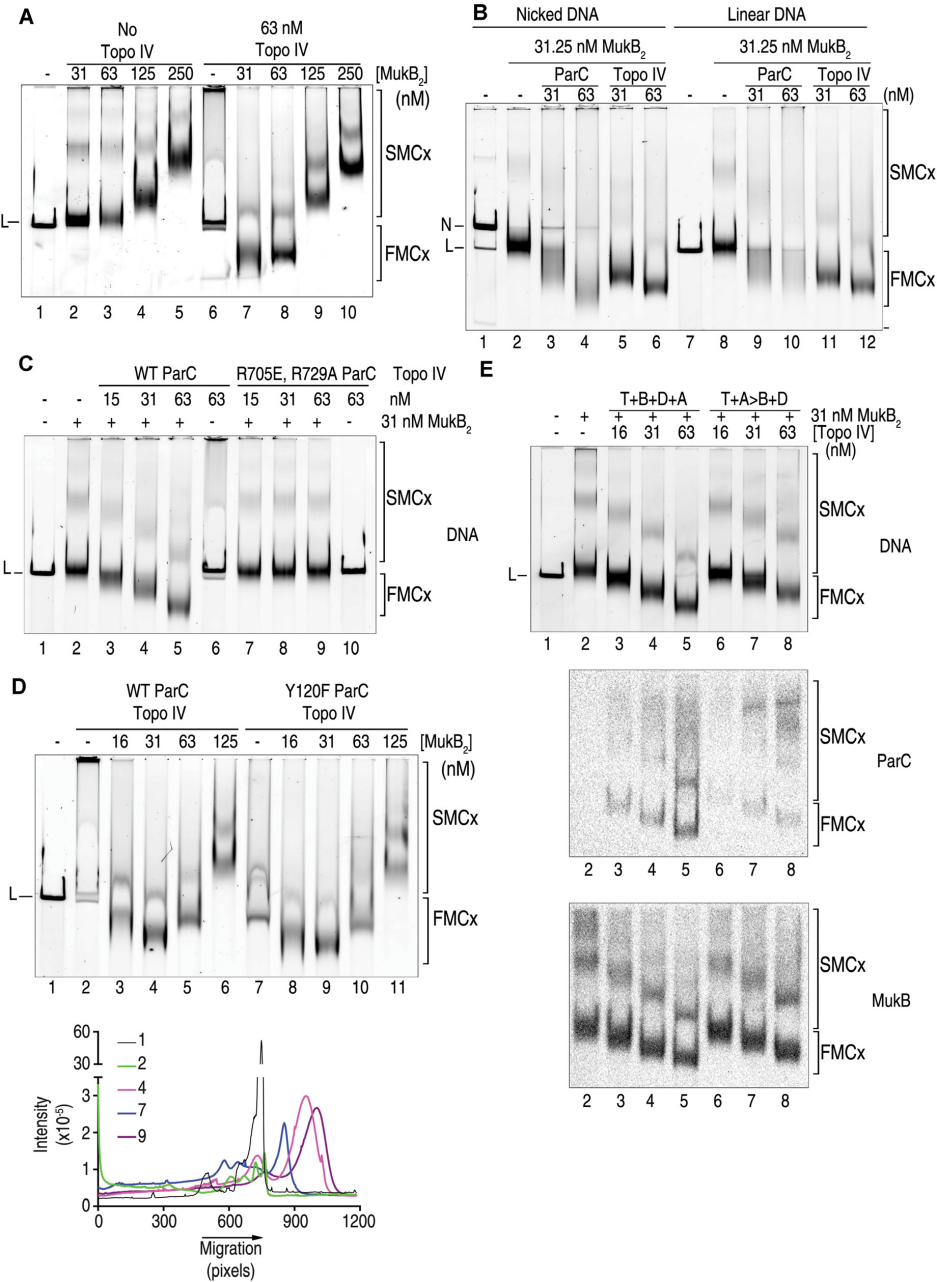
Condensation of linear DNA by MukB and Topo IV. (**A**) MukB and Topo IV together can condense linear plasmid DNA. The indicated concentrations of MukB and Topo IV were incubated in DNA condensation reaction mixtures containing linear plasmid DNA for 5 min and the products were analyzed by agarose gel electrophoresis as described under Materials and Methods. (**B**) Linear DNA condensation requires Topo IV holoenzyme. In the presence of MukB, the indicated concentrations of either the ParC subunit of Topo IV or Topo IV were incubated in DNA condensation reaction mixtures containing either singly nicked (lanes 1–6) or linear (lanes 7–12) plasmid DNA for 5 min and the products were analyzed by agarose gel electrophoresis as described under Materials and Methods. (**C**) The MukB-Topo IV interaction is required for linear DNA condensation. The indicated concentrations of Topo IV reconstituted with either wild-type ParC or the non-MukB-interacting variant ParC R705E R729A (4) were incubated in DNA condensation reaction mixtures in the presence of MukB and linear plasmid DNA for 5 min and the products were analyzed by agarose gel electrophoresis as described under Materials and Methods. (**D**) The catalytic activity of Topo IV is not required for linear DNA condensation. Topo IV (62.5 nM) reconstituted with either wild-type ParC or the catalytically inactive variant ParCY120F was incubated in DNA condensation reaction mixtures in the presence of linear plasmid DNA and the indicated concentrations of MukB and the products were analyzed by agarose gel electrophoresis as described under Materials and Methods. Top panel, a representative agarose gel. Bottom panel, densitometric tracing of the indicated gel lanes from the gel in the top panel. (**E**) Closing the Topo IV N gate before it is exposed to DNA reduces the amount of Topo IV on the DNA. Top panel, lanes 2–5. The indicated concentrations of MukB and Topo IV were incubated in DNA condensation reaction mixtures with linear plasmid DNA in the presence of 5 mM Mg-AMP⋅PNP for 10 min. Lanes 6–8, the DNA condensation reactions were performed in two steps: First Topo IV was pre-incubated in the presence of 5 mM Mg-AMP⋅PNP for 5 min, MukB was then added and the incubation continued for 5 min. Reaction mixtures were split in half and reaction products were analyzed by electrophoresis through two agarose gels as described under Materials and Methods. Middle and bottom panels, Southwestern transfers were then performed: one gel was blotted for ParC (middle panel) and the other gel for MukB (bottom panel).

Incubation of increasing concentrations of MukB with a linear 11-kb plasmid DNA with blunt ends resulted in the generation of DNA–protein complexes of decreasing mobility on agarose gels compared to the linear DNA itself (Figure 2A and Supplementary figure S2A, lanes 1–5), as expected for complexes of increasing mass. On the other hand, in the presence of Topo IV, concentrations of MukB in the range of 31-63 nM generated protein–DNA complexes that migrated significantly faster than the linear DNA (Figure 2A and Supplementary Figure S2A, compare lane 1 with lanes 7 and 8). Higher concentrations of MukB resulted in slower moving species (Figure 2A and Supplementary Figure S2A, compare lane 1 with lanes 9 and 10). As we have argued previously (6,23), the appearance of these faster moving complexes indicates that irrespective of the fact that Topo IV and MukB are both present on the DNA (see below), the DNA itself has become condensed to the extent that the protein–DNA complex migrates faster in the gel than the DNA alone.

We characterized the nature of these fast-moving DNA–protein complexes (Figure 2B–E and Supplementary Figure S2B–D). Whereas the ParC subunit of Topo IV alone was capable of generating condensed DNA complexes with MukB on nicked DNA ((23), and Figure 2B and Supplementary Figure S2B), it was much less effective than holoenzyme Topo IV on linear DNA (Figure 2B and Supplementary Figure S2B, compare lanes 9 and 10 with lanes 11 and 12). This observation is not surprising given that although ParC is a DNA-binding protein, alone it cannot create a DNA crossover as Topo IV does.

Formation of the fast-moving DNA complex was not simply an additive effect of Topo IV and MukB binding to separate regions of the DNA, but a synergistic one requiring the interaction between the two proteins (Figure 2C). MukB with Topo IV reconstituted from wild-type ParC and ParE subunits formed the fast-moving complex (Figure 2C, lanes 1–5), whereas with Topo IV reconstituted from wild-type ParE and ParC R705E R729A, a variant that no longer interacts with MukB (4), did not (Figure 2C, lanes 6–10).

Because our assays did not contain ATP, it seemed clear that the catalytic activity of neither MukB nor Topo IV was required for the DNA condensation described here. To confirm this, we compared the ability of a catalytically inactive Topo IV, where the active site tyrosine had been mutated to a phenylalanine (ParC Y120F Topo IV), to that of the wild-type enzyme (Figure 2D, upper panel). Both Topo IV preparations cooperated with MukB to form the fast-moving condensed DNA–protein complex (Figure 2D, upper panel, compare lane 1 with lanes 4 and 9). Interestingly, Topo IV itself could condense the DNA (Figure 2D, upper panel, compare lane 1 with lanes 2 and 7). We have determined that this condensation is the result of Topo IV stabilizing crossovers in the DNA (see below). Furthermore, the catalytically inactive Topo IV was more efficient a DNA condensing agent than the wild-type Topo IV, as judged by the mobility of the protein–DNA complexes (Figure 2D, lower panel, compare lanes 4 and 9). [Note, at these high concentrations, wild-type Topo IV has a tendency to aggregate, hence the observed DNA in the well of lane 2 in Figure 2D. However, comparison of the mobility of the material that does run into the gel between lanes 2 and 7 shows a significant difference in the mobility of the protein–DNA complexes, as does the mobility of the MukB-Topo IV complexes formed with wild-type (lane 4) and ParC Y120F Topo IV (lane 9). This difference can also be observed in Supplementary Figure S2E below, where there is less aggregation of the wild-type Topo IV].

If trapping of a DNA crossover was part of the role of Topo IV in producing the condensed linear DNA species with MukB, then pre-incubation of Topo IV with AMP⋅PNP should close the N gate and prevent crossover trapping, as described above in Figure 1B. As shown in Figure 2E, such a pre-incubation consistently produced DNA–protein complexes that were less condensed than those generated with Topo IV that had not been exposed to AMP⋅PNP before DNA (Figure 2E, upper panel, compare lanes 3–5 with lanes 6–8, and Supplementary Figure S2C). The reason for this effect became apparent when the protein–DNA complexes were probed with antibodies for the presence of MukB and ParC (Figure 2E, middle, and lower panels, and Supplementary Figure S2D). Whereas the amount of MukB present on the DNA was unaffected by the presence or absence of Topo IV, with or without pre-incubation of the Topo IV with AMP⋅PNP (Figure 2E, lower panel, and Supplementary Figure S2D), pre-incubation with AMP⋅PNP reduced the amount of Topo IV in the fast-moving protein–DNA complex by 65% on average (Figure 2E, middle panel, and Supplementary Figure S2D).

To better understand the effect of Topo IV DNA condensation by itself, we compared on one gel the protein–DNA complexes formed with linear DNA and different combinations of wild-type Topo IV, ParC Y120F Topo IV, MukB, and MukB D697K D745K E753K (MukB^triple^), a MukB variant that does not interact with ParC (33) (Supplementary Figure S2E). In the absence of AMP⋅PNP, wild-type Topo IV retards the mobility of the linear DNA slightly (Supplementary Figure S2E, compare lanes 1 and 4), whereas ParC Y120F Topo IV does the same, but also manifests a faster moving band (Supplementary Figure S2E, compare lanes 1 and 5), indicating some DNA condensation. In both cases, however, addition of MukB generates a hyper-condensed DNA band (Supplementary Figure S2E, compare lanes 1, 4, and 6 and 1, 5, and 8), the formation of which is dependent on the interaction between MukB and Topo IV (Supplementary Figure S2E, compare lanes 6 and 7 and lanes 8 and 9), as also shown in Figures 1 and 2. When AMP⋅PNP is added to both wild-type and ParC Y120F Topo IV subsequent to exposure to DNA, the extent of formation of fast-moving, condensed protein–DNA complexes increases (compare lanes 4 and 5 to lanes 13 and 14), confirming that the formation of this complex by Topo IV itself arises because of trapping of DNA crossovers. Nevertheless, even in the artificial situation where AMP⋅PNP is present to close the Topo IV N gate, addition of MukB to both wild-type and ParC 120F Topo IV results in the formation of a faster moving, more defined protein–DNA complex (Supplementary Figure S2E, compare lane 15 with lane 13 and lane 17 with lane 14), the formation of which requires the interaction between ParC and MukB (Supplementary Figure S2E, compare lane 16 with lane 15 and lane 18 with lane 17).

**Figure 3.**
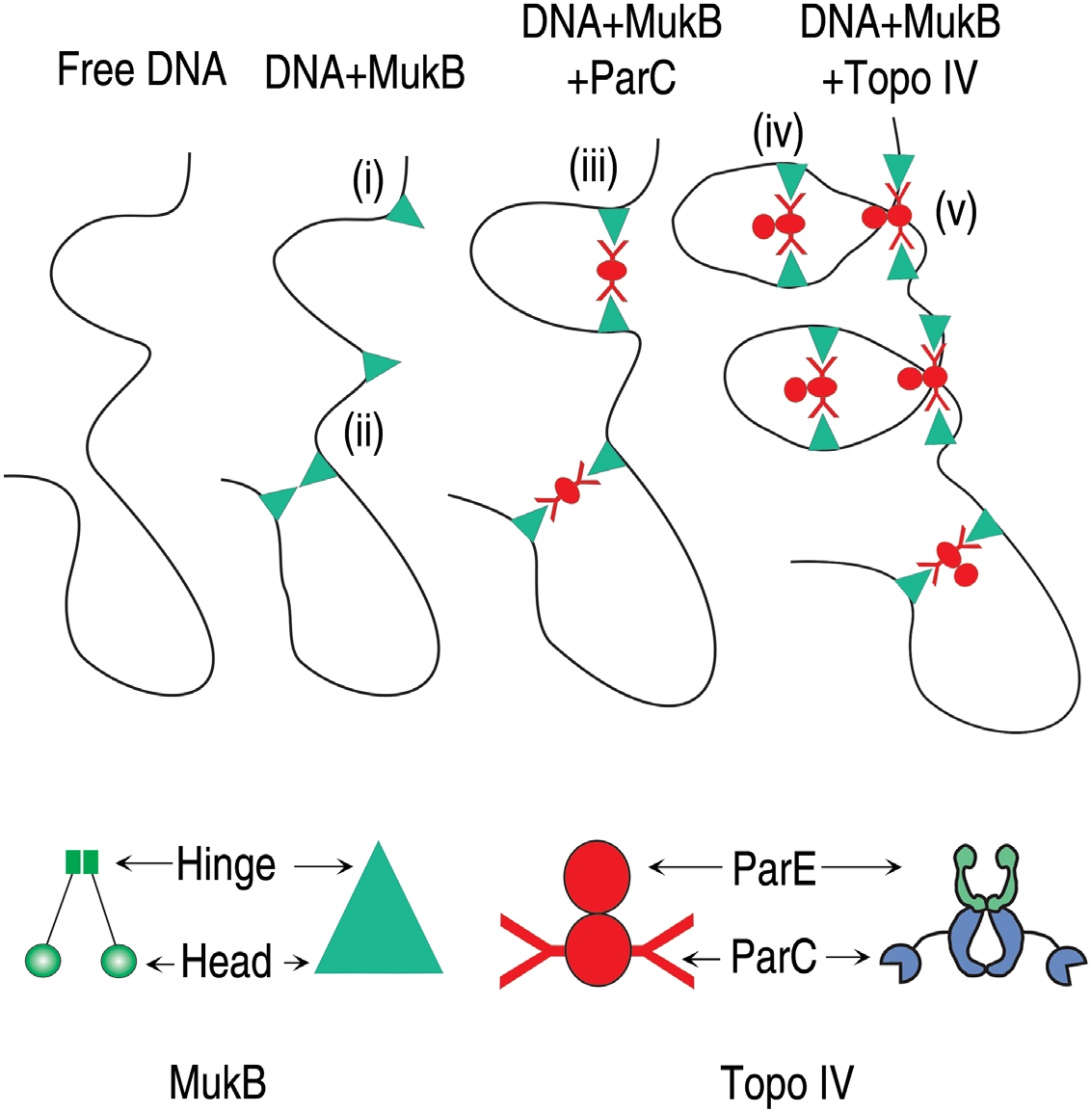
Model for MukB and Topo IV condensation of linear DNA. (i) MukB bound to DNA. (ii) DNA loop stabilization by hinge-hinge interactions between MukB dimers. (iii) and (iv) ParC alone and Topo IV can bridge distant MukB dimers on the DNA to stabilize loops. (v) Topo IV stabilizes crossovers in the DNA, organizing it into a series of DNA loops.

In sum, these results suggest that with linear DNA, whereas MukB can clearly bind and presumably can associate via hinge-hinge interactions between dimers to form loops, as we have shown previously (23), these loops are likely large and are insufficient to generate the change in topology observed for the hyper-condensed DNA–protein complex (Figure 3, i and ii). ParC alone can aid the formation of the large DNA loops by bridging MukB dimers (Figure 3, iii), but only Topo IV can introduce additional loops in the DNA by trapping a DNA crossover, thus generating numerous topological domains that can be acted on further by MukB dimers bridged by Topo IV to generate the hyper-condensed form (Figure 3, iv and v). MukB alone can generate a fast-moving, condensed DNA form on nicked plasmid DNA because the circular nature of the DNA amplifies the generation of isolated topological domains. We consider why MukB alone fails to generate a condensed protein–DNA complex with linear DNA in the Discussion.

### MukB suppresses DNA crossover capture by topo IV

In a Topo IV reaction, crossover stabilization by binding of two DNA segments is followed by DNA cleavage, strand passage, re-ligation, and release of the T segment. The hydrolysis of ATP is believed to keep the cycle moving forward and to recycle the enzyme for the next round of catalysis. Although we did not use ATP in our reactions, the presence of ATP in vivo would presumably keep Topo IV cycling between these steps. In such a scenario, a short-lived crossover stabilization by Topo IV may not be sufficient for stimulation of condensation by MukB. However, the greater condensation of DNA in the linear DNA–MukB–Topo IV complex by Topo IV with a catalytically inactive ParC subunit compared to the wild-type (Figure 2D) suggested that MukB might be modulating either the activity of or interaction with DNA by Topo IV.

**Figure 4.**
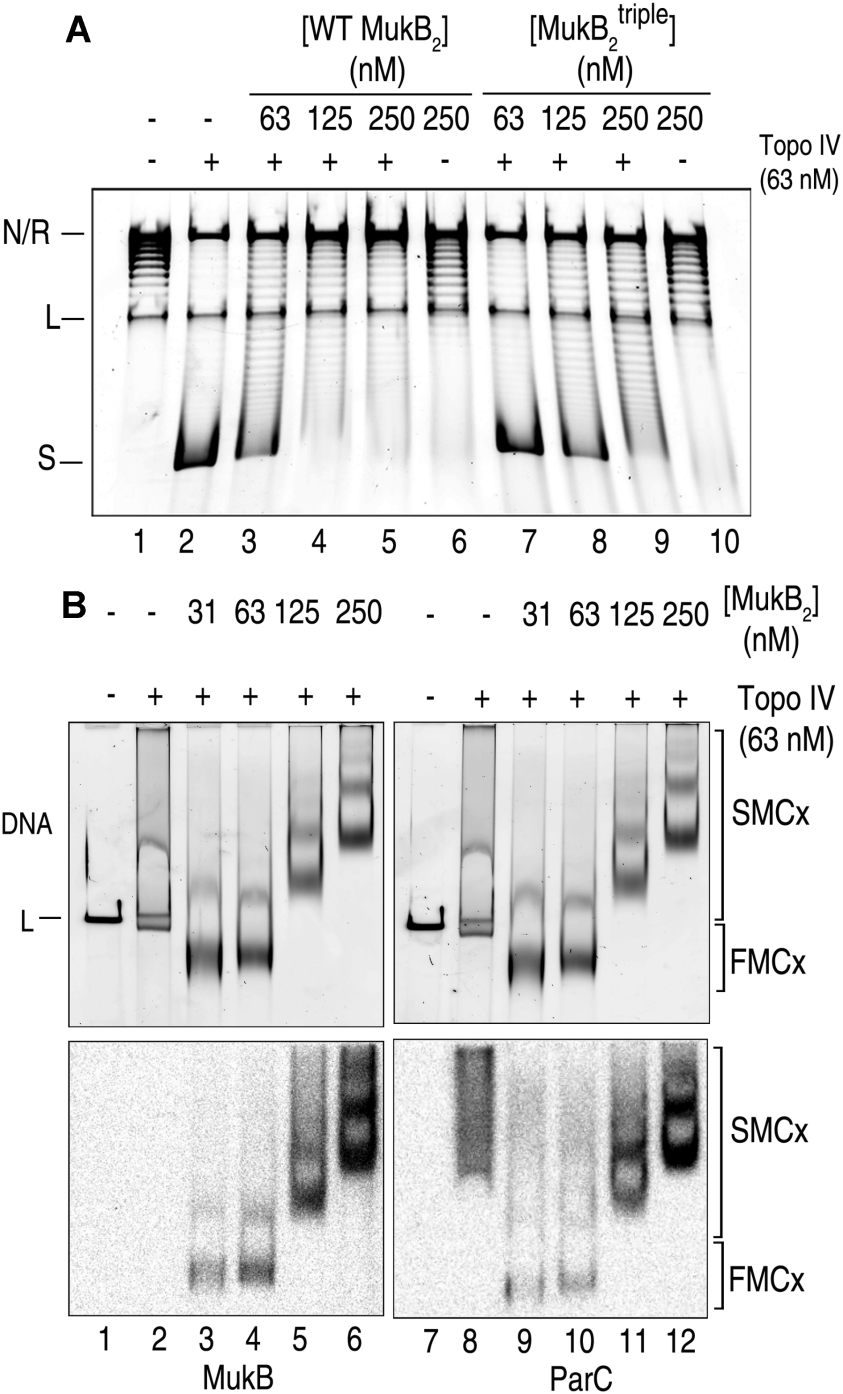
MukB inhibits Topo IV trapping of DNA crossovers. (**A**) Induction of negative supercoils by Topo IV is inhibited by MukB. The indicated concentrations of ParC Y120F Topo IV and either wild-type MukB (lanes 3–6) or the non-ParC-interacting variant MukB D697K D745K E753K (MukB^triple^) (lanes 7–10) were bound to singly-nicked plasmid DNA for 5 min at 37°C, *E. coli* DNA ligase was then added and the incubation continued for another 5 min to seal the nick. Samples were deproteinized and the resulting induced supercoils present were analyzed by agarose gel electrophoresis as described under Materials and Methods. R, relaxed; S., supercoiled. (**B**) MukB binding to DNA does not inhibit Topo IV binding to DNA. Top panels, the indicated concentrations of MukB and Topo IV were incubated in DNA condensation reaction mixtures containing linear DNA for 5 min. The reaction mixtures were split in half and the reaction products were analyzed by electrophoresis through two agarose gels. Lower panel, southwestern blots of the gels shown in the top panel for the presence of MukB (left) and ParC (right).

We first examined the effect of MukB on the ability of ParC 120F Topo IV to trap negative crossovers (Figure 4). This phenomenon is revealed when ParC Y120F Topo IV is bound to a singly-nicked DNA, the nick is then sealed with *E. coli* DNA ligase, the proteins removed by proteinase K digestions, and the DNA then electrophoresed through an agarose gel (Figure 4A). Topo IV induces negative supercoils in the DNA because of trapping right-handed crossovers in a stoichiometric manner (Figure 4A, compare lanes 1 and 2). Addition of MukB to this reaction inhibited the ability of Topo IV to trap crossovers as revealed by the decline in negative supercoiling when both proteins were present (Figure 4A, compare lanes 3–5 with lane 2). Although MukB alone can induce negative supercoils in relaxed DNA (23), the extent of supercoiling observed is not as great as that observed with Topo IV and requires the presence of chloroquine in the gel for detection. For comparison, Supplementary Figure S3A shows the effect of MukB alone on the DNA. The observed inhibitory effect was not the result of MukB preventing Topo IV from binding the DNA. Interaction between the two proteins was required (Figure 4A, compare lanes 7–9 with lanes 2–4) and southwestern blotting of a typical reaction gel for the presence of MukB and ParC showed increasing amounts of both proteins on the DNA as induction of negative supercoils was inhibited (Figure 4B). Because the non-ParC interacting MukB^triple^ variant (apparent *K*_D_ = 30 nM) actually binds DNA better than the wild-type protein (apparent *K*_D_ = 120 nM) (Supplementary Figure S3B), decreased binding of MukB^triple^ to DNA cannot account for the differential effect observed in Figure 4B compared to the wild type. We therefore considered that it was possible that MukB actually inhibited Topo IV activity rather than stimulated it, as has been reported (4,5,33,41). This issue is difficult to approach *in vitro* given that Topo IV is active at concentrations that are much lower than those required to observe the interaction between the two proteins and the effect of Topo IV itself, not its catalytic activity, on DNA topology. We thus turned to assessing Topo IV activity *in vivo* in the presence and absence of MukB as an assay.

**Figure 5.**
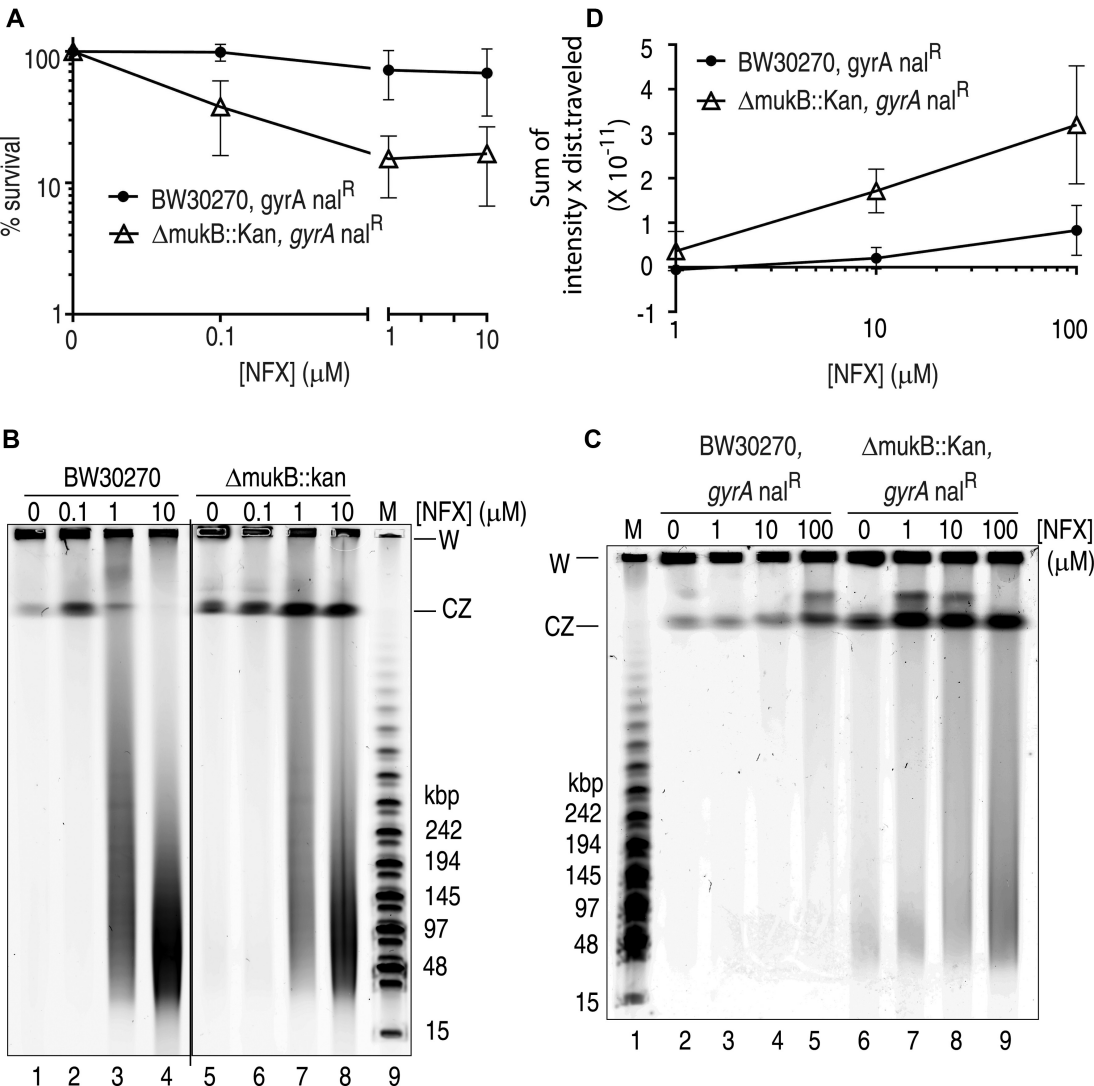
*mukB* deletion increases sensitivity to norfloxacin and suppresses Topo IV-catalyzed DNA cleavage in a *gyrA^nalR^* background. (**A**) Norfloxacin sensitivity of BW30270 *gyrA^nalR^* and BW30270 *gyrA^nalR^ΔmukB*. Cells were grown at 25ºC in LB medium to mid-log phase, treated with the indicated concentrations of norfloxacin for 30 min, the drug was washed out, cells were then plated on LB plates that did not contain norfloxacin, and survival scored as described under Materials and Methods. NFX, norfloxacin. (**B**) Norfloxacin-induced DNA cleavage is unaffected by *mukB* deletion in a wild-type *gyrA* background. BW30270 and BW30270 *ΔmukB* cells were grown and treated with norfloxacin as above. After washing, cells were formed in agarose plugs, and treated with SDS and proteinase K and the DNA subjected to pulsed-field gel electrophoretic analysis as described under Materials and Methods. W, well; CZ, compression zone. (**C**) *mukB* deletion increases norfloxacin-induced DNA cleavage in a *gyrA^nalR^* background. Pulsed-field gel electrophoretic analysis of DNA cleavage in BW30270 *gyrA^nalR^* and BW30270 *gyrA^nalR^ΔmukB* cells treated with the indicated concentrations of norfloxacin as described above and under Materials and Methods. (**D**) *mukB* deletion increases norfloxacin-induced DNA cleavage in a *gyrA^nalR^* background. Plot (mean and standard deviation, *n* = 3) of the densitometric intensity in any given pixel multiplied by the distance from the well for each lane of three independent gels including the one shown in panel C.

The obvious readout of Topo IV activity in the cell is the extent of topological linkage between the sister chromosomes. But because inactivation of either MukB (7) or Topo IV (15,20) cause defects in chromosome conformation, we used norfloxacin-induced chromosome cleavage as a measure of Topo IV activity (42). We first assessed the sensitivity of cells to norfloxacin, a quinolone that inhibits both DNA gyrase and Topo IV (22), in the presence and absence of a *mukB* deletion, which causes cells to grow somewhat more slowly than their isogenic parent (Supplementary Figure S4A). These cells also carried the S83L mutation in the GyrA subunit of DNA gyrase, which renders that enzyme resistant to the quinolone family of drugs including nalidixic acid and norfloxacin (43), so that the assay was measuring the effect of the drug on Topo IV (Figure 5A). The BW30270 *gyrA^nal^^R^ ΔmukB* strain was consistently about 5-fold more sensitive to killing by 1 and 10 μM norfloxacin treatment than the parent strain BW30270 *gyrA^nal^^R^*. Previous observations have shown that *ΔmukB* strains had significantly increased sensitivity to novobiocin [the inhibitor of the ATPase activity of DNA gyrase (44) and Topo IV (18,19)] and slightly increased sensitivity to norfloxacin (45,46). Sensitivity was related to activity of the MukBEF condensin, was not related to multidrug efflux, and is thought to be a result of increased demand for type II topoisomerase activity during DNA replication (46). The increased sensitivity to norfloxacin of BW30270 *gyrA^nalR^ ΔmukB* is consistent with these prior observations and with the idea that the presence of MukB in complex with Topo IV constrains the activity of the latter protein. Thus, when MukB is absent, more active Topo IV is present on the chromosome, making the strain more sensitive to the poison norfloxacin, as well as satisfying the increased demand for topoisomerase activity.

To expand this observation, we assessed the extent of DNA cleavage directly by pulsed-field gel electrophoresis (Figure 5B-D and Supplementary Figure S4B and C). Cells in early log phase were exposed to varying concentrations of norfloxacin for 30 min and then prepared for PFGE. Treatment with proteinase K and SDS will convert the open complex of Topo IV-norfloxacin-chromosomal DNA to a frank double-stranded DNA break and these DNA fragments will, unlike the circular chromosome or replicating intermediates thereof, migrate into the gel.

In the absence of the *gyrA^nal^^R^* mutation, both the wild-type and *ΔmukB* strain exhibited similar extents of DNA cleavage (Figure 5B and Supplementary Figure S4B). This result is expected because DNA gyrase is the major target of the quinolones in *E. coli* (47) and also demonstrates that MukB has no direct effect on DNA gyrase activity, consistent with our previous observation that MukB does not interact with the GyrA subunit of DNA gyrase (4). On the other hand, in the presence of the *gyrA^nal^^R^* mutation, *ΔmukB* cells displayed significantly greater amounts of DNA cleavage than the wild type (Figure 5C, D, and Supplementary Figure S4C, compare lanes 7–9 with lanes 3–5). It should be noted that this DNA cleavage assay is stoichiometric with respect to Topo IV. That is, a DNA fragment that enters the gel has been generated by cleavage of two molecules of Topo IV frozen in an open complex by the action of norfloxacin. Thus, generation of decreasingly-sized DNA fragments indicates an increasing amount of DNA cleavages by Topo IV. In order to scale the overall extent of cleavage properly in Figure 5C, the intensity of any particular pixel in the lane on the gel was multiplied by the number of pixels migrated and these products were summed to give the total cleavage (Figure 5D).

**Figure 6.**
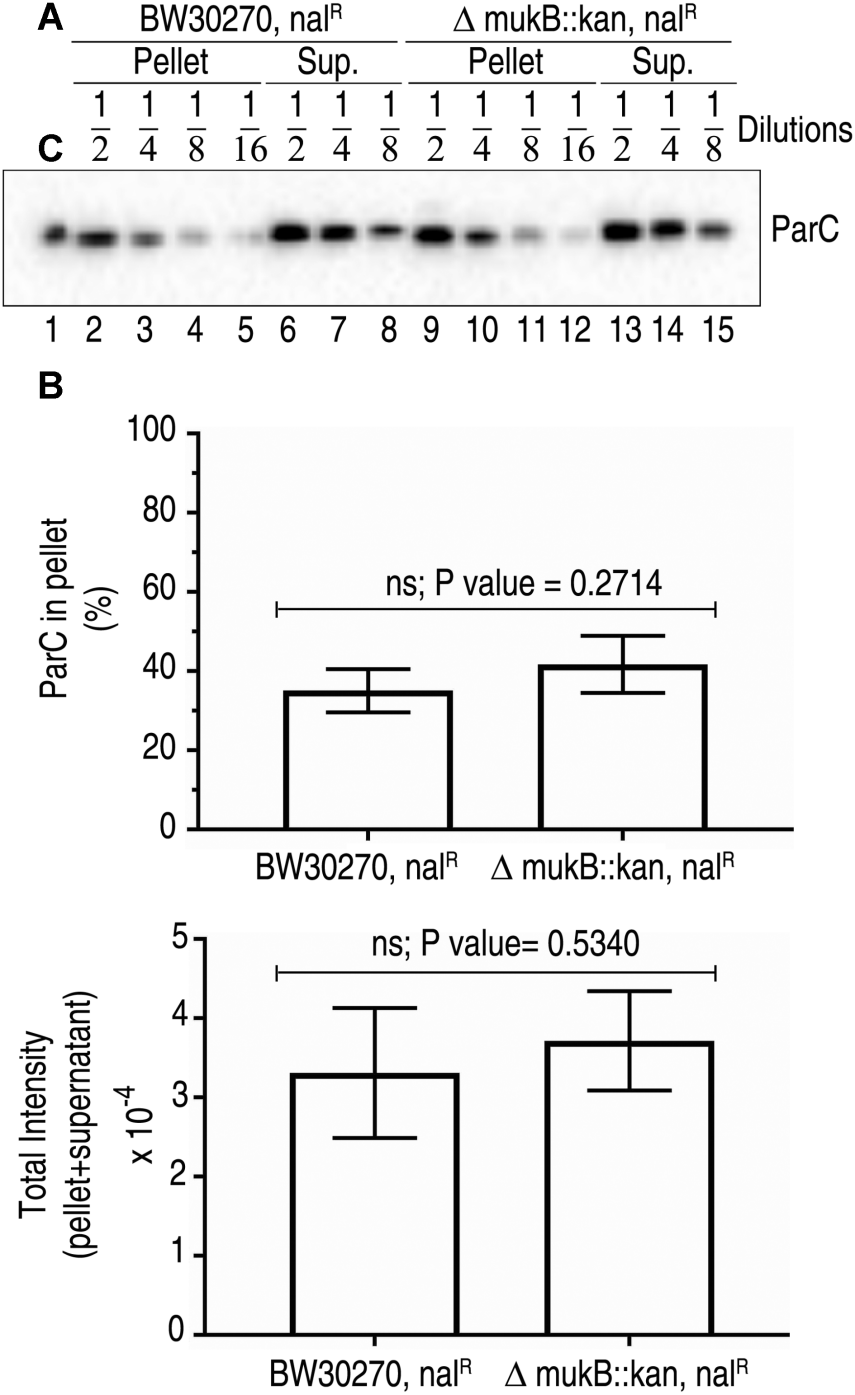
Distribution of Topo IV between soluble and DNA-bound fractions in BW30270 *gyrA^nalR^* and BW30270 *gyrA^nalR^ ΔmukB* cells. (**A**) BW30270 *gyrA^nalR^* and BW30270 *gyrA^nalR^ ΔmukB* cells were grown to mid-log phase in LB medium at 25ºC, harvested, lysed with lysozyme and Brij, and fractionated into soluble and pellet fractions by centrifugation at 100 000 × g. The soluble and pellet fractions were subjected to SDS-PAGE and the gel western blotted for the ParC subunit of Topo IV as described under Materials and Methods. (**B**) *top panel*, fraction of DNA-bound ParC in BW30270 *gyrA^nalR^* and BW30270 *gyrA^nalR^ ΔmukB*. Shown is the mean and standard deviation (*n* = 3). *Bottom panel*, Total ParC present in the DNA-bound pellet and free in the supernatant for BW30270 *gyrA^nalR^* and BW30270 *gyrA^nalR^ ΔmukB*. Shown is the mean and standard deviation (*n* = 3). There are no statistically significant differences between the values shown for the two strains in either case (Student's *t*-test).

This observation suggested that the presence of MukB limited the catalytic activity of Topo IV on the chromosome. Of course, there was also a possible trivial explanation in that there might simply be more Topo IV on the DNA in the *ΔmukB* cells to deal with possible entanglements in the absence of proper chromosome organization. To address this possibility we examined the distribution of Topo IV between the DNA-bound chromatin fraction and the soluble cytoplasm in the BW30270 *gyrA^nalR^* and BW30270 *gyrA^nalR^ ΔmukB* strains grown to early log phase (Figure 6).

Equivalent amounts of cells (by O.D._600_) were lysed in the presence of lysozyme and Brij 58 and the soluble fraction separated from the chromatin fraction by centrifugation. The pellet was resuspended by sonication. Equivalent amounts of the two fractions from each strain were then subjected to SDS-PAGE and the gel blotted for the presence of the ParC subunit of Topo IV (Figure 6A). We found that there was about 19% more Topo IV present in the chromatin fraction in the BW30270 *gyrA^nalR^ ΔmukB* strain compared to the BW30270 *gyrA^nalR^* strain and about 12% more total Topo IV in the former strain compared to the latter (Figure 6B). Neither of these differences were statistically significant (by the Student's t-test). Furthermore, given the stoichiometric nature of the cleavage reaction detailed in Figure 5 and discussed above, this slight increase in Topo IV on the DNA cannot account for the significantly greater DNA cleavage observed in the BW30270 *gyrA^nalR^ ΔmukB* strain compared to the BW30270 *gyrA^nalR^* strain (Figure 5B-D and Supplementary Figure S4A and B).

Another possible explanation for our observations was that if the nalidixic acid-resistant gyrase was less active than the wild type, demand for Topo IV during decatenation might be increased, leading to more of the enzyme engaged at any time with the chromosomal DNA. Previous observations had, in fact, shown that several nalidixic acid-resistant DNA gyrases were less active than wild type (48,49), although the GyrA S83L enzyme was not (49), nor was a GyrA S83A gyrase (50). To be certain on this issue, we purified GyrA S83L, reconstituted it with GyrB, and tested both its supercoiling activity and resistance to norfloxacin compared to the wild type enzyme (Supplementary Figure S5). GyrA S83L gyrase had essentially identical activity to the wild type (Supplementary Figure S5, top panel, compare lanes 7–11 with lanes 2–6) and was about 16-fold more resistant to inhibition by norfloxacin than the wild type enzyme (Supplementary Figure S5, bottom panel, compare lanes 8–13 to lanes 2–7). Thus, this possibility seems unlikely and we therefore conclude that in the MukB-Topo IV complex on DNA *in vivo*, the catalytic activity of Topo IV is suppressed.

### The ParC subunit of topo IV inhibits the MukB ATPase

**Figure 7.**
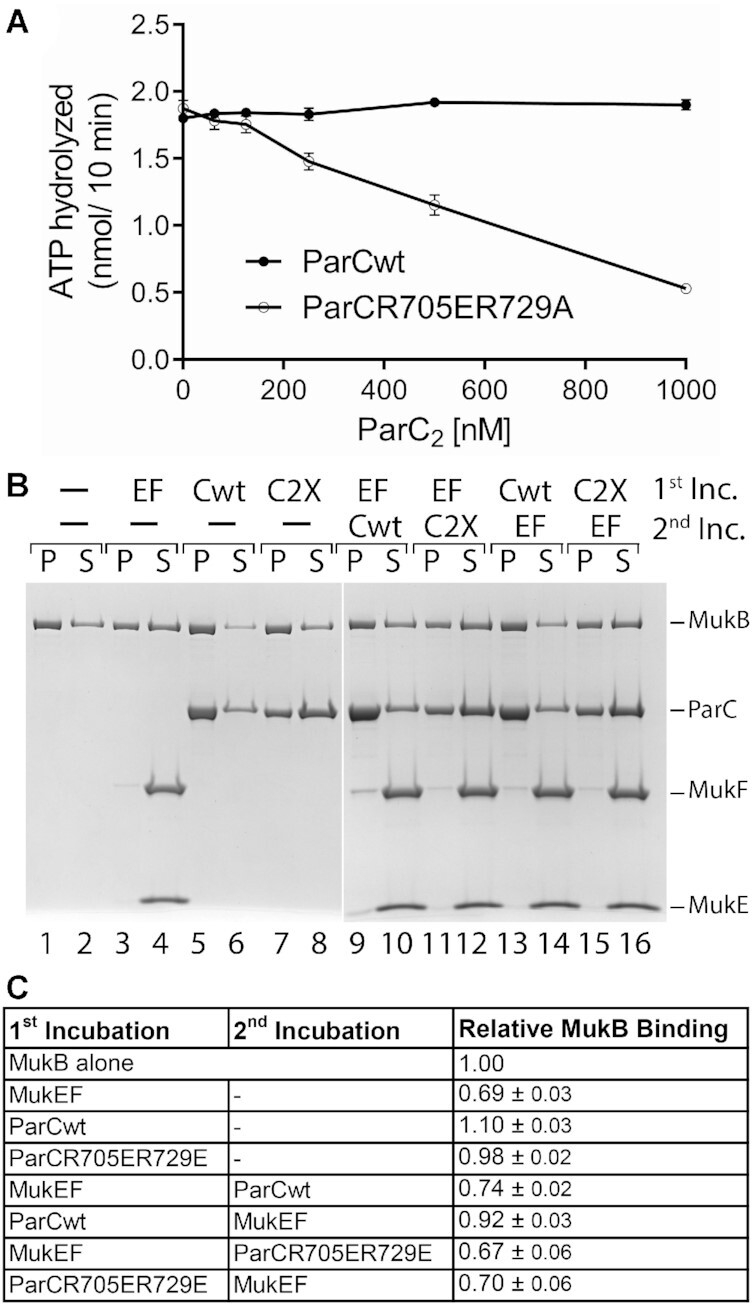
ParC inhibits the MukF-stimulated ATPase activity of MukB and stabilizes it on DNA. (**A**) MukBF ATPase activity was measured as described under Materials and Methods in the presence of either no additional proteins or either the ParC R705ER729A or wild-type ParC subunit of Topo IV. Shown is the mean and standard deviation (*n* = 3). (**B**) MukB-DNA pull-down as described under Materials and Methods. Lanes 1–8, MukB–DNA magnetic beads, were incubated with either MukEF, wild-type ParC (Cwt), or ParC R705ER729A (C2X) for 5 min at 37°C, the beads were pulled down, and the pellet and supernatant analyzed by SDS-PAGE. Lanes 9–16. Two step incubations: Reactions containing MukB-DNA magnetic beads and additional proteins as indicated, were incubated in two steps (the second step was also 5 min at 37°C), the beads were pulled down, and the pellet and supernatant analyzed by SDS-PAGE. Lanes 1–8 and lanes 9–16 are two different representative gels. (**C**) Relative amount of MukB binding to the DNA on the beads in the experiments described in *panel B*. Shown is the mean and standard deviation (*n* = 3).

Given the effect of MukB on Topo IV, we asked the obverse question as to whether Topo IV had any effect on MukB activities. MukB is an ATPase, an activity that would presumably be required for any form of loop extrusion or translocation on DNA. The MukB ATPase is stimulated significantly by the kleisin, MukF and, unlike most SMC proteins (1), is DNA-independent (31,51). Because it is the ParC subunit of Topo IV that interacts with MukB (4,5) and Topo IV is itself an ATPase, we asked if ParC affected the MukBF ATPase activity. As shown in Figure 7A, this proved to be the case. Inhibition of MukBF ATPase activity by ParC was dependent on the interaction between the two proteins, as the non-interacting ParC R705E R729A variant had no effect (Figure 7A). We conclude that in the Topo IV-MukB complex each protein inhibits the activity of the other.

It has been demonstrated that MukF inhibits DNA binding by MukB (9). The results described above suggested that Topo IV might stabilize MukB on DNA. To address this question, MukB was bound to linear DNA coupled to magnetic beads. Free MukB was removed and the MukB–DNA-beads incubated with either MukEF or the ParC subunit of Topo IV. Following pull down of the beads on a magnet, the distribution of MukB in the pellet (on DNA) and free in the supernatant was determined (Figure 7B). The addition of MukEF destabilized the MukB, whereas the addition of ParC stabilized it (Figure 7B, compare lanes 3 and 4 to lanes 5 and 6), as measured by the fraction of MukB remaining on the DNA after pull down (Figure 7C). We then asked if ParC stabilization was effected even in the presence of MukEF. To do so we used a two-stage incubation. The MukB-DNA-beads were first incubated with either MukEF or ParC for 5 min followed by addition of the other protein and an additional 5 min incubation and then pull down. MukB binding to the DNA was destabilized if MukEF were added before ParC, whereas if ParC was present first, MukB DNA binding was preserved (Figure 7B and C, compare lanes 9 and 10 to lanes 13 and 14). ParC stabilization of MukB required the interaction between the two proteins. ParC R705ER729A, which does not interact with MukB (4), failed to stabilize MukB on the DNA (Figure 7B and C, compare lanes 5 and 6 with lanes 7 and 8; and lanes 13 and 14 with lanes 15 and 16). Thus our results indicate that MukB and Topo IV form a stable complex on the DNA in which their activities are suppressed. It seems likely that the result is the formation of a stable loop in the DNA.

## DISCUSSION

The condensin MukBEF and the chromosome decatenating enzyme Topo IV are clearly essential for the proper management of chromosome conformation in *E. coli*. The ability of MukB to mechanically condense DNA in an ATP-dependent fashion has been established in single molecule assays (52) and the decatenating activity of Topo IV demonstrated both *in vitro* (17) and *in vivo* (16). Thus, the demonstration that they interacted (4,5) led to suggestions that MukB was likely stimulating the decatenation activity of Topo IV *in vivo* (53–56). Indeed, the original reports describing the interaction between the two proteins detailed the stimulation of Topo IV superhelical DNA relaxation activity, although there was disagreement as to whether the decatenation activity of the enzyme was stimulated similarly (4,5). Subsequent work from this laboratory argued that MukB stimulated intramolecular activities of Topo IV, such as superhelical DNA relaxation and DNA knotting, but not intermolecular activities, such as decatenation of multiply-linked DNA dimers that represented catenated sister chromosomes at the terminal stages of DNA replication (33).

Here we have demonstrated that when in complex together, the two enzymes actually exhibit inhibitory activity toward each other: The ParC subunit of Topo IV inhibits the MukF-stimulated ATPase activity of MukB (Figure 7) and MukB inhibits the DNA cleavage activity of Topo IV, as well as affects how it interacts with DNA (Figures 4 and 5). A key difference between the current observations and the previous ones is that in this report, MukB and Topo IV are present at roughly equal stoichiometry. Previous observations of stimulation (4,5,33) were based on MukB being in vast excess to Topo IV and thus, we suspect, were reporting a target site size effect whereby MukB on the DNA substrates was serving as an attractor for Topo IV (stimulation was dependent on the interaction between the two proteins (4,33)) that was present at low concentration in the reaction mixtures). MukB and Topo IV are present at similar concentrations in cells growing slowly on minimal medium [450–600 nM MukB, 450–900 nM ParC, and 300–600 nM ParE (30,55,57)]. Using a pull-down assay with immobilized MukB and added ParC, we find the *K*_D_ of the interaction to be 115 nM (dimer to dimer, Supplementary Figure S6), a value likely to be lower *in vivo* because of significant molecular crowding. Thus, one can certainly expect there to be significant interaction between them *in vivo*. The reduction in crossover capture by Topo IV in the presence of MukB (Figure 4) is consistent with inhibition of its ability to modulate DNA topology. We therefore argue that MukB does not stimulate the decatenation activity of Topo IV *in vivo*, instead Topo IV acts as a brake on MukB and *vice versa* and the two enzymes in complex form a stable platform to maintain condensed loops of chromosomal DNA. Indeed, we could demonstrate that a ParC–MukB complex was more stable on the DNA than a MukB–MukEF complex (Figure 7).

Surprisingly, given the preference of the enzyme for relaxing positive supercoils (58,59), we have found that the DNA crossover stabilized by Topo IV under these conditions was right-handed. We have investigated the nature of this effect and its relationship to Topo IV activity. These results will be reported elsewhere.

The use of linear DNA as a substrate for condensation in this study has allowed us to mimic long contiguous stretches of genomic DNA that need to be condensed by MukB/MukBEF. In contrast to assays with nicked DNA, MukB was unable to form complexes with linear DNA that migrated faster than the free DNA, suggesting that MukB/MukBEF alone may not be able to condense genomic DNA. The presence of Topo IV restored MukB-condensation of linear DNA. Although the ATP analog AMP⋅PNP did not have a significant effect on MukB DNA binding, a substantial decrease of MukB DNA condensation was observed upon preincubation of Topo IV with AMP⋅PNP. This observation was surprising in that ATP binds the ParE subunit, whereas MukB is known to interact with the ParC subunit. Moreover, ParC alone could stimulate condensation on a nicked substrate, but Topo IV holoenzyme was required to observe such an effect with linear DNA. The observed reduction in stimulation of MukB DNA condensation in the presence of AMP⋅PNP could be because of the inability of Topo IV to interact with a second DNA segment. It is likely Topo IV generates a looped structure because of its interaction with two distant segments of a linear DNA. As MukB prefers condensation of a circular over a linear DNA, such a looped structure may be favorable for MukB action resulting in the stimulation of DNA condensation. Thus, Topo IV may assist MukBEF in condensing genomic DNA by stabilizing a looped structure by interaction with two distant segments of DNA.

Topo IV has been demonstrated to co-localize with MukBEF clusters by live-cell imaging (54) and photoactivated localization microscopy (55). These clusters concentrate at *oriC*. Disruption of the MukB-ParC interaction by over-expression of the C-terminal domain of ParC (which interacts with the hinge region of MukB (4,5)) caused a delay in the segregation of newly-replicated origin regions, leading to the conclusion that MukB was stimulating Topo IV decatenation activity. Loading of Topo IV to the chromosome has been proposed to be an intricate balance between the interaction of MukBEF with Topo IV and the interaction of MatP with MukBEF (56), which causes dissociation of the latter from the chromosome. This latter interaction excludes MukBEF from the *ter* region of the chromosome, and, presumably, to some extent, Topo IV. On the other hand, a comparison (42) of Topo IV ChIP data with sites of norfloxacin-induced DNA cleavage (NorfIP) in *gyrA^nalR^* cells, thus marking sites of Topo IV activity, indicated that most sites of Topo IV binding (by ChIP) were not sites of activity (by NorfIP) and vice versa. The strongest activity site for Topo IV was the *dif* locus, which is located in the *ter* region, and cleavage at this site was only reduced by about 30% in the presence of a *mukB* deletion.

Consistent with our conclusion presented above, these observations suggest that co-localization of MukB with Topo IV doesn’t necessarily imply direct stimulation of Topo IV activity. The MukB-Topo IV interaction might attract Topo IV to the DNA, but that enzyme may or may not be active and stimulation of Topo IV activity by MukB may only reflect increased loading of the former on the chromosome. We also note that norfloxacin-induced TUNEL labeling in synchronized populations of *gyrA^nalR^* cells was restricted to late in the cell cycle, when replication was essentially complete (60), consistent with this activity occurring in the *ter* region. Thus, the MukB-Topo IV interaction may focus Topo IV activity to the *ter* region because of expulsion of the inhibitory MukB by MatP.

How do our observations relate to the likely ability of MukBEF to extrude DNA loops and/or translocate on DNA? Clearly, loop extrusion by MukBEF would require an active ATPase activity. Inhibition of the MukB ATPase by Topo IV implies that in a MukB-Topo IV complex on DNA, any motor function of MukB is severely attenuated. Thus Topo IV may effectively function like mammalian CTCF, which is thought to stop loop extrusion catalyzed by cohesion (61).

Our DNA condensation assay shows that MukB alone can alter the topology of DNA. Neither closure of the ATP heads nor ATPase activity is required to observe the fast-moving protein–DNA complexes. This suggests that under the conditions of our assay, MukB is making stable contact with the DNA in at least two locations. The lack of a significant effect on closure of the MukB heads with AMP⋅PNP suggests that the two head domains in a MukB dimer might bind DNA independently. The hinge region also binds DNA (23). If our assays reflect an intermediate in a loop extrusion process, this capture of a loop by MukB in the absence of ATP presumably reflects the necessity of directionality in the orientation of the loop in order to afford a net positive process when it is coupled to the ATP hydrolytic cycle. This may be why MukB alone fails to form a fast-moving complex with linear DNA. With circular DNA substrate, as with a nicked plasmid or a linear DNA circularized by the crossover-capture activity of Topo IV, nearby segments of DNA are, to some extent, more constrained in their location relative to a bound protein than on a linear DNA, thus making capture of the ‘next’ segment of DNA more deterministic.

## DATA AVAILABILITY

Original gel data and densitometric tracings have been presented in the manuscript after labeling in Adobe Photoshop. Raw gel images and ATPase data are held by the authors and are available on request.

## Supplementary Material

gkab1027_Supplemental_File
