# Supplementary material for: The MukB-topoisomerase IV interaction mutually suppresses their catalytic activities
Source: Nucleic Acids Res. 2021 Nov 8;50(5):2621–34. doi: 10.1093/nar/gkab1027 (PMC8934648; doi:10.1093/nar/gkab1027)
Supplement: gkab1027_Supplemental_File [file gkab1027_supplemental_file.pdf]

SUPPLEMENTARY INFORMATION

The MukB-Topoisomerase IV Interaction Mutually Suppresses Their Catalytic Activities

Rupesh Kumar, Soon Bahng, and Kenneth J. Marians*

Molecular Biology Program, Memorial Sloan Kettering Cancer Center, 1275 York Avenue, New York, NY 10065

*Correspondence. E-mail: kmarians@sloankettering.edu; tel: (212) 639-5890

Supplementary Figures 1-6 and Figure Legends

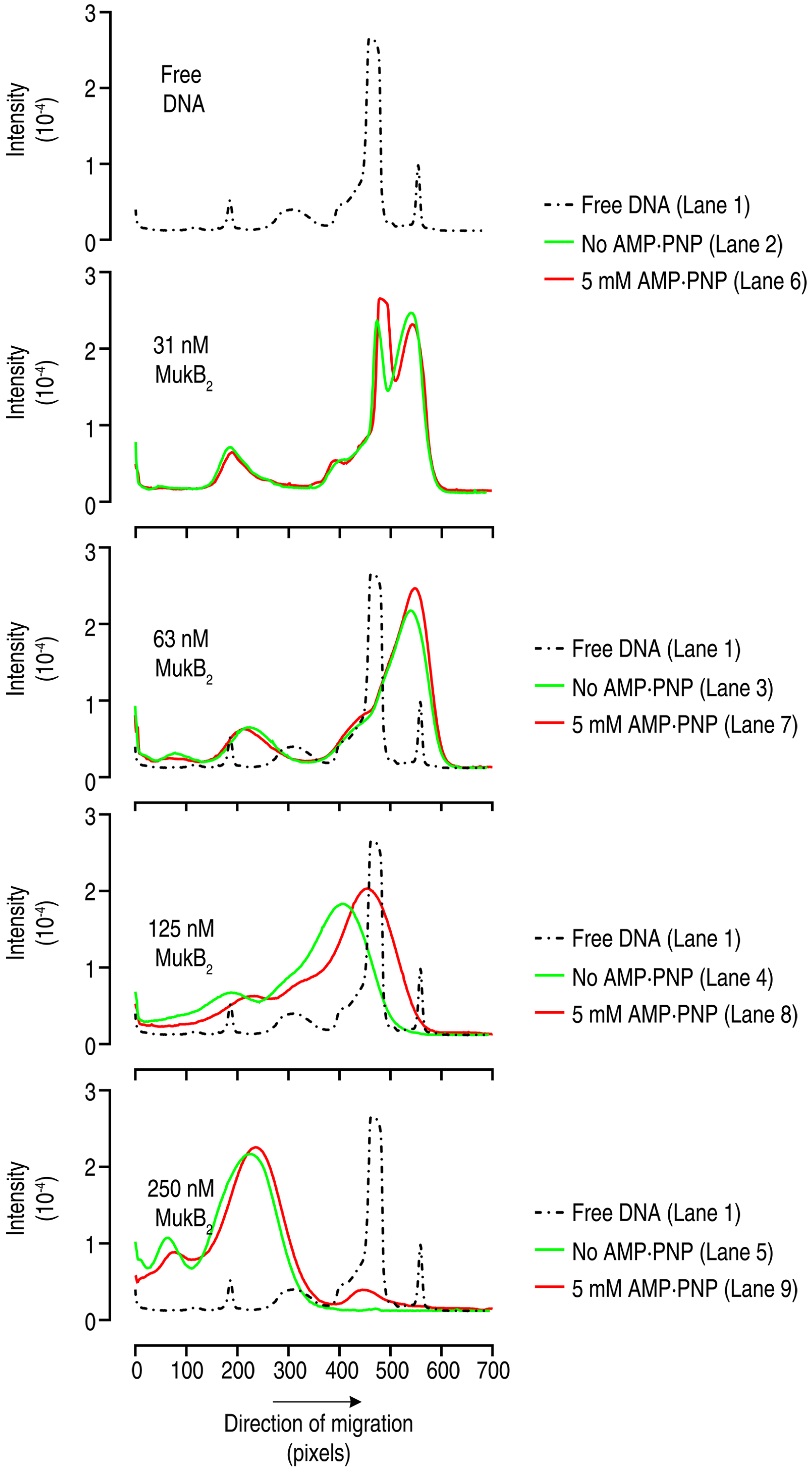

Supplementary Figure 1(A). Densitometric tracings of the lanes in the gel shown in Figure 1A.

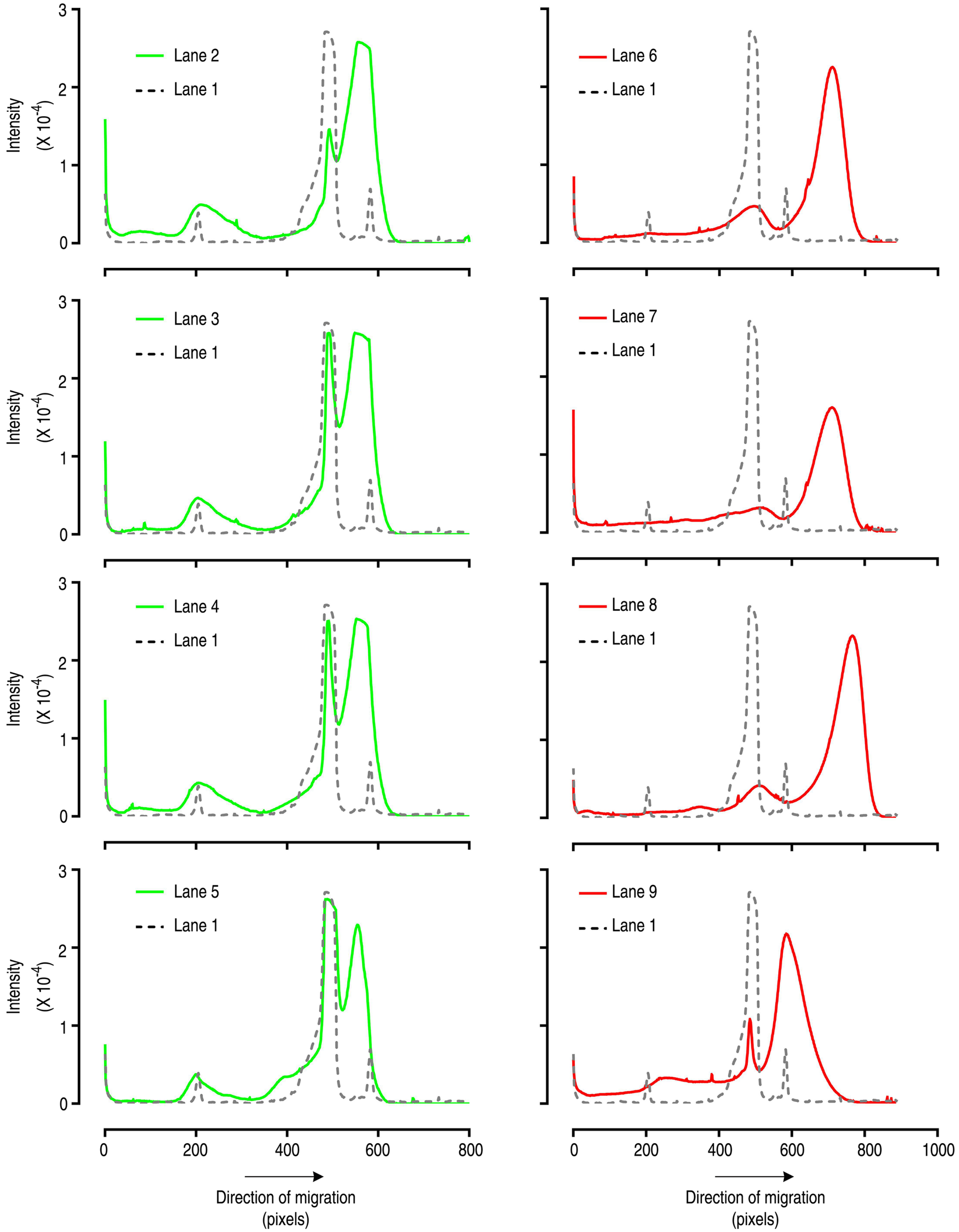

Supplementary Figure 1(B). Densitometric tracings of the lanes of the gel shown in Figure 1B.

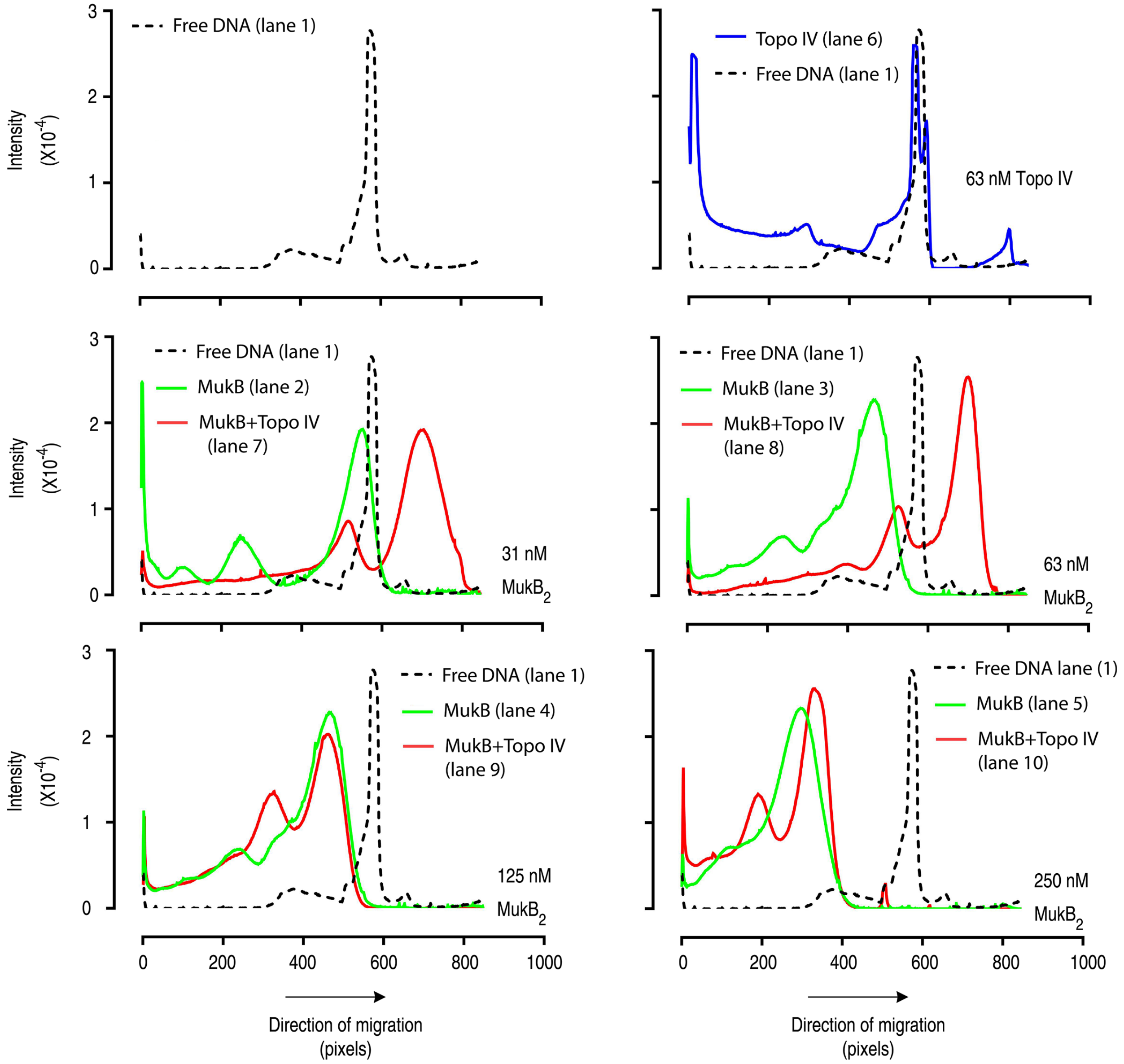

Supplementary Figure 2(A). Densitometric tracings of the lanes of the gel shown in Figure 2A.

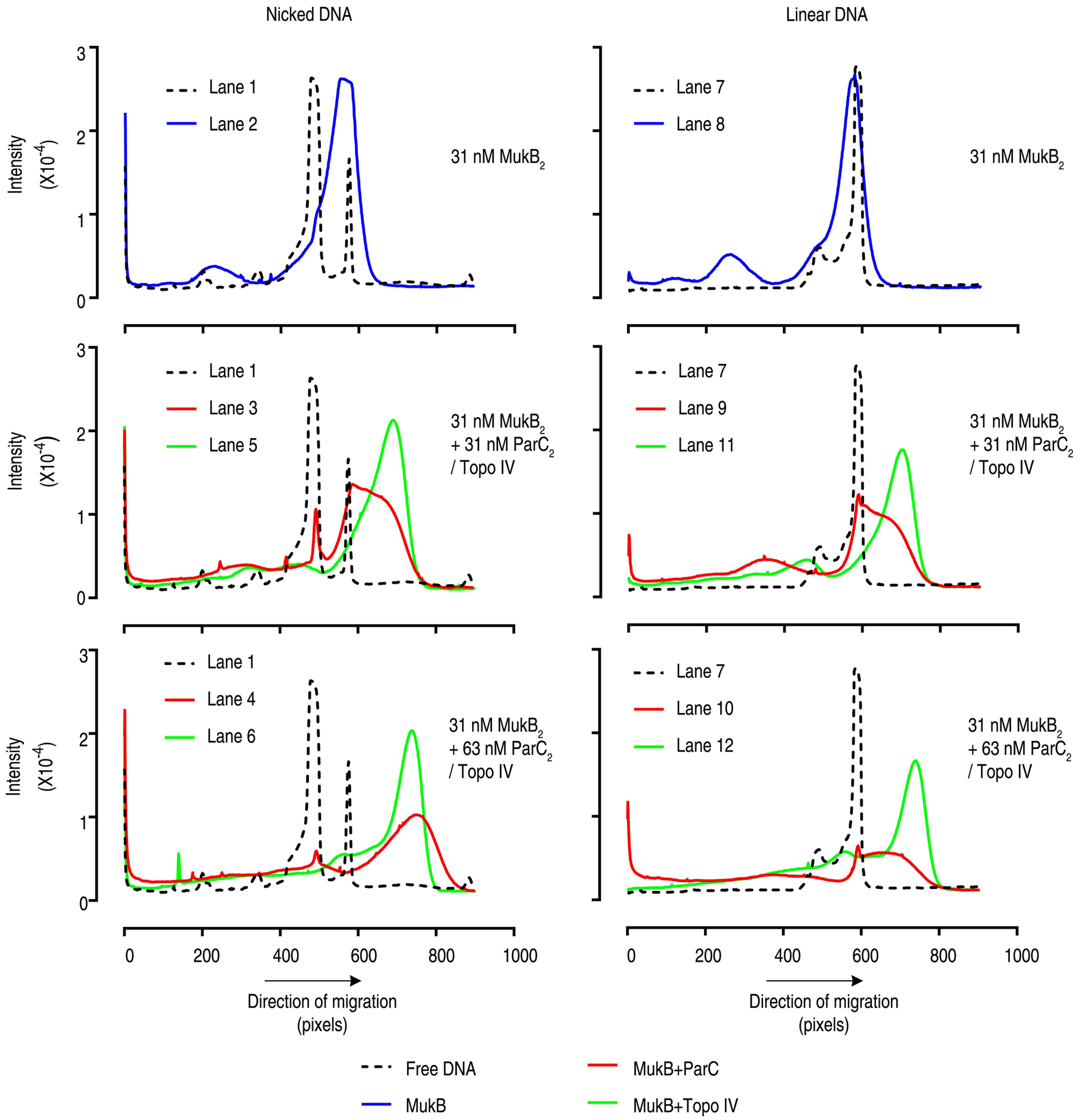

Supplementary Figure 2(B). Densitometric tracings of the lanes of the gel shown in Figure 2B.

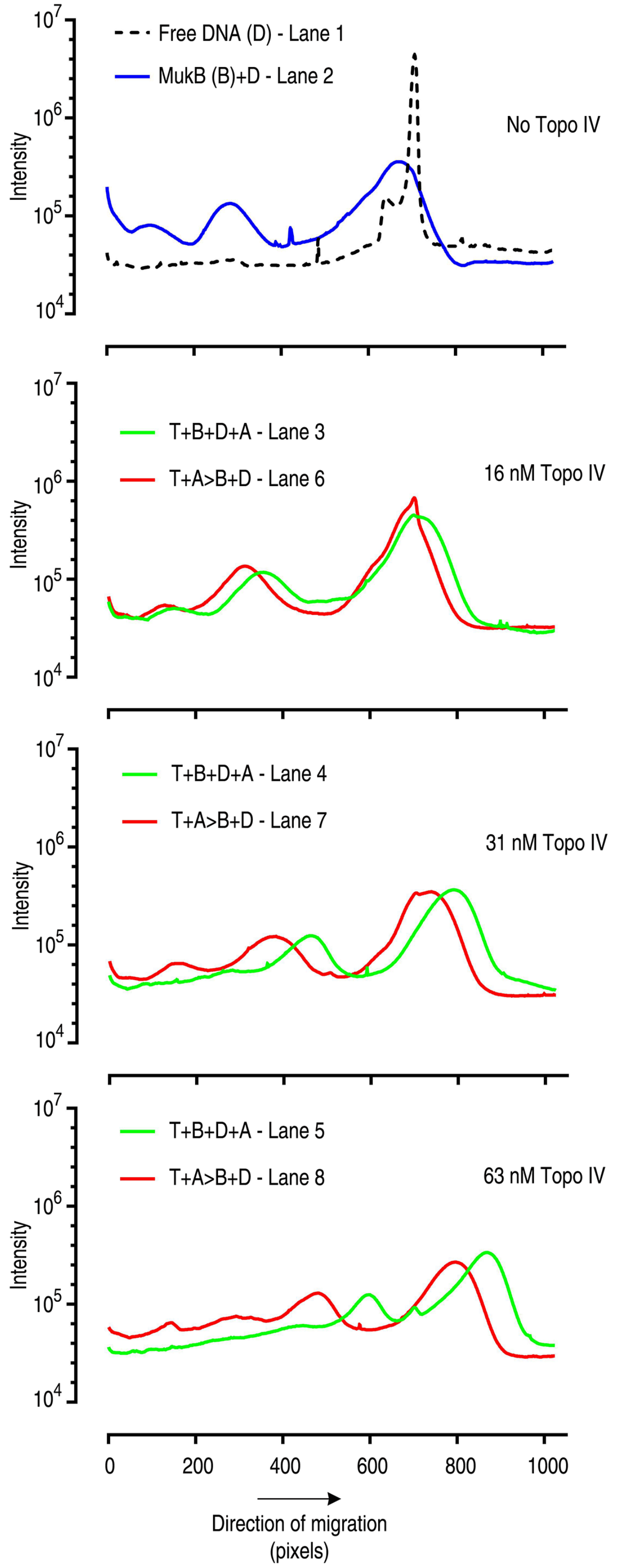

Supplementary Figure 2(C). Densitometric tracings of the lanes of the gel shown in the upper panel of Figure 2E.

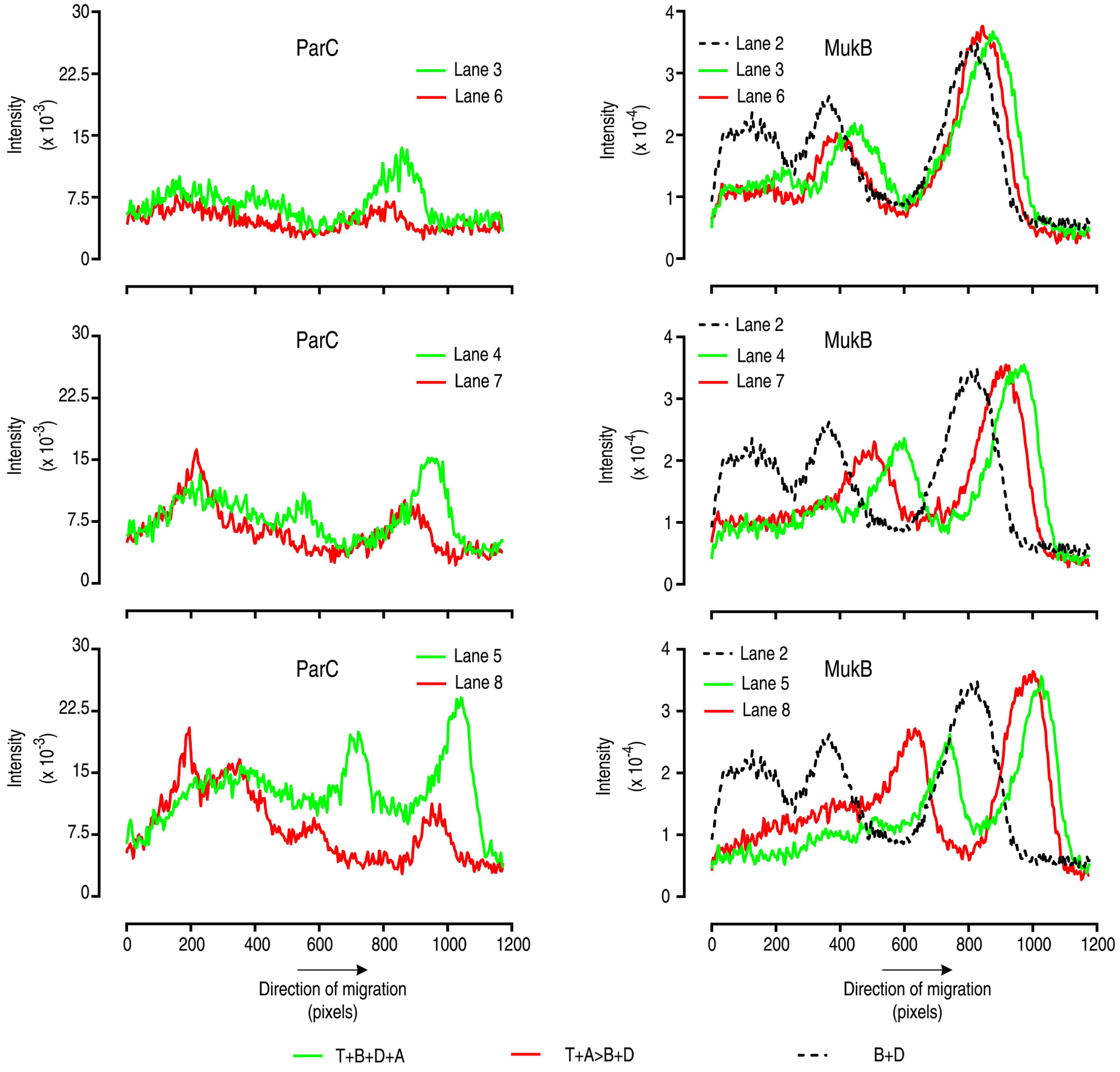

Supplementary Figure 2(D). Densitometric tracings of the lanes of the gel shown in the middle and lower panels of Figure 2E.

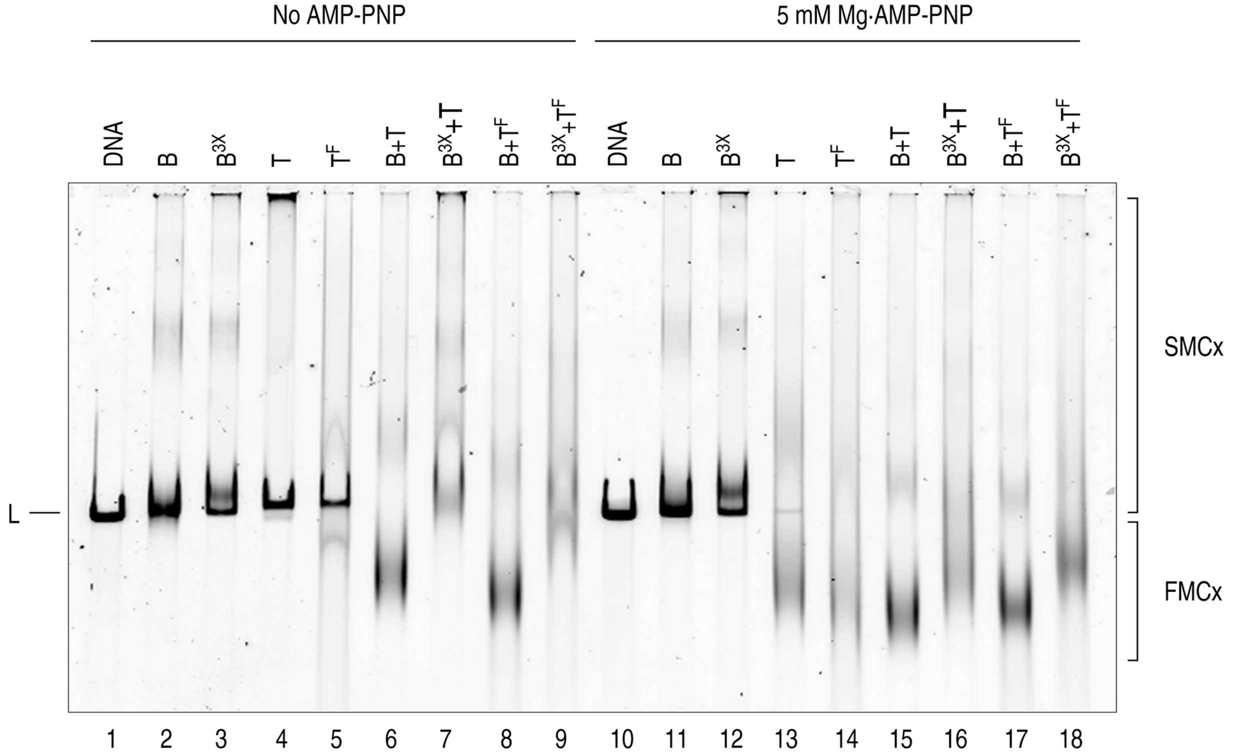

Supplementary Figure 2(E). Generation of fast-moving protein-DNA complexes with linear DNA in the presence and absence of AMP-PNP. The indicated combinations of wild-type MukB (B), MukB D697K D745K E753K (B^{3X}), wild-type Topo IV (T), and ParCY120F Topo IV (T^F), with MukB's at 31.25 nM and Topo IV's at 62.5 nM were incubated with linear pCG09 DNA either in the presence or absence of 5 mM Mg-AMP-PNP for 5 min at 37 °C and the protein-DNA complexes formed analyzed by agarose gel electrophoresis as described under Materials and Methods. L, linear DNA; SMCx, slow-moving complex; FMCx, fast-moving complex.

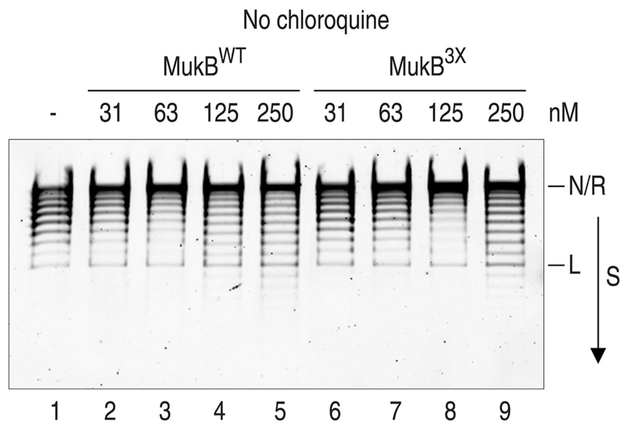

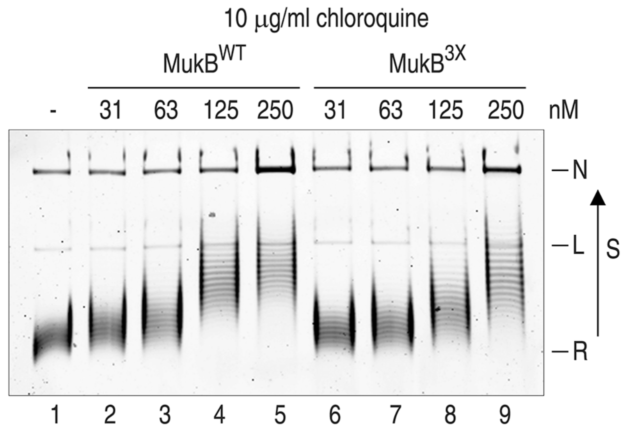

Supplementary Figure 3(A). Induction of negative supercoils in DNA by MukB. The indicated concentrations of either wild-type (WT) or D697K D745K E753K ($MukB^{3X}$) MukB were bound to singly-nicked plasmid DNA for 5 min at 37 °C, E. coli DNA ligase was then added and the incubation continued for another 5 min to seal the nick. Samples were deproteinized and analyzed by agarose gel electrophoresis in either the presence or absence of chloroquine as described under Materials and Methods. N, nicked; R, relaxed; S., supercoiled. The direction of the arrow indicates increasing number of negative supercoils.

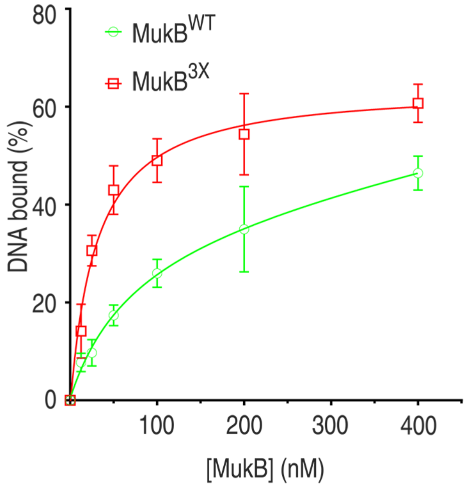

Supplementary Figure 3(B). DNA-binding activity of wild-type (WT) and D697K D745K E753K ($MukB^{3X}$) MukB. DNA binding reaction mixtures (20 μl) containing 50 mM HEPES-KOH (pH 7.5), 10 mM DTT, 20 mM KCl, 0.5 mM $Mg(OAc)_2$, 5 nM [5′-^{32}P] 50mer duplex DNA, and either no, 12.5 nM, 25 nM, 50 nM, 100 nM, 200 nM, and 400 nM of either wild-type or D697K D745K E753K MukB were incubated for 15 min at 37 °C. Reaction mixtures were then diluted with 100 μl wash buffer [50 mM HEPES-KOH (pH 7.5), 20 mM KCl, 0.5 mM $Mg(OAc)_2$] that had been warmed to 37 °C and filtered through Millipore 0.45 μm HAWP nitrocellulose filters that had been soaked in wash buffer. The filters were washed three times with 1 ml of wash buffer, dried, and the radioactivity retained determined by liquid scintillation counting. Shown is the mean and standard deviation (n=3). 50mer DNA sequence: Top strand: 5′-ATCTCGGGCTATTCTTTTGATTTATAAGGGATTTTGCCGATTTCGGAACC-3′; bottom strand: 5′-GGTTCCGAAATCGGCAAAATCCCTTATAAATCAAAAGAATAGCCCGAGAT-3′.

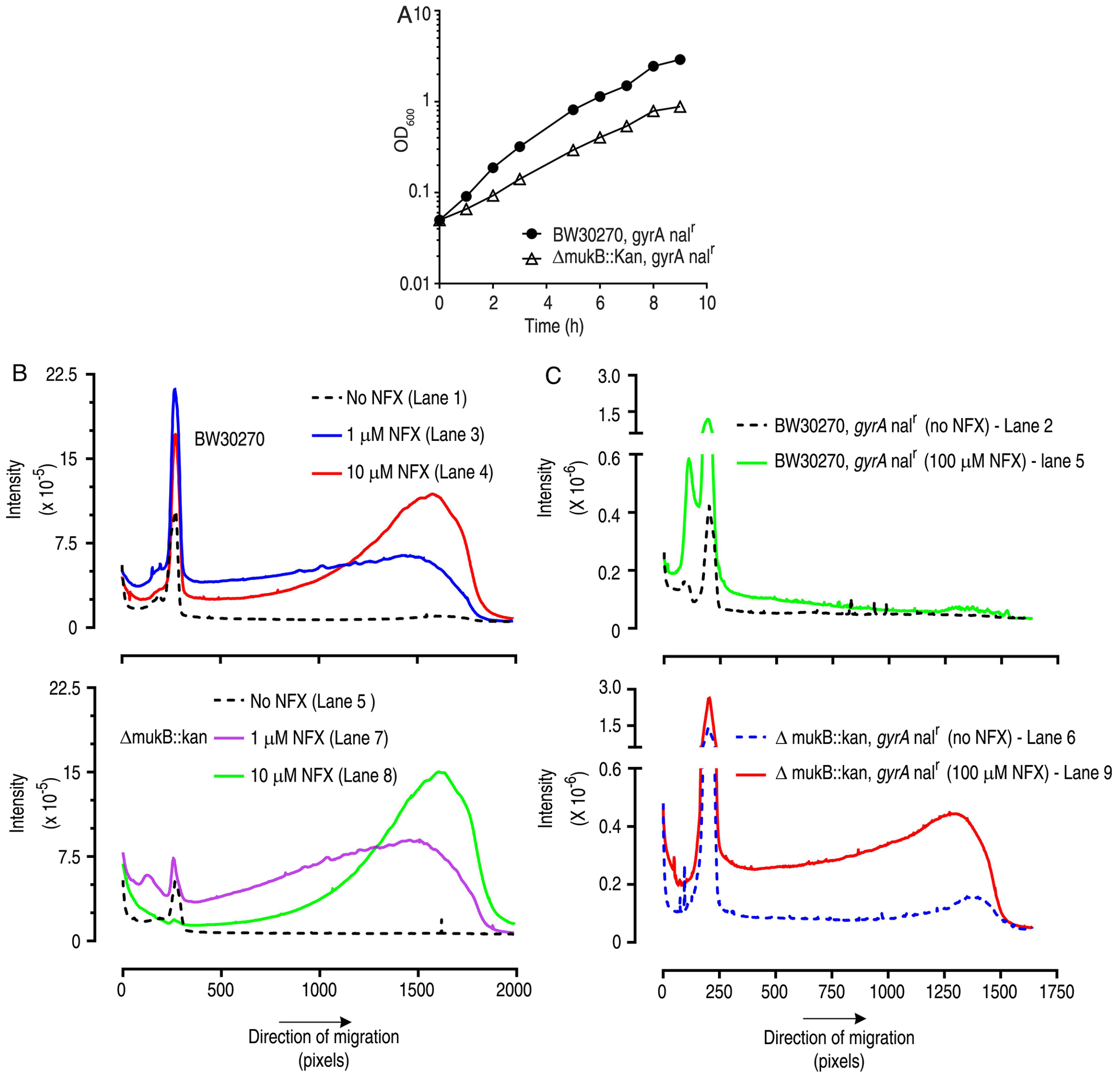

Supplementary Figure 4. (A) Growth curves of BW30270 *gyrA*^{nalR} and BW30270 *ΔmukB::kan gyrA*^{nalR} in LB medium at 25 °C. **(B)** Densitometric tracings of the lanes of the gel shown in Figure 5B. **(C)** Densitometric tracings of the lanes of the gel shown in Figure 5C.

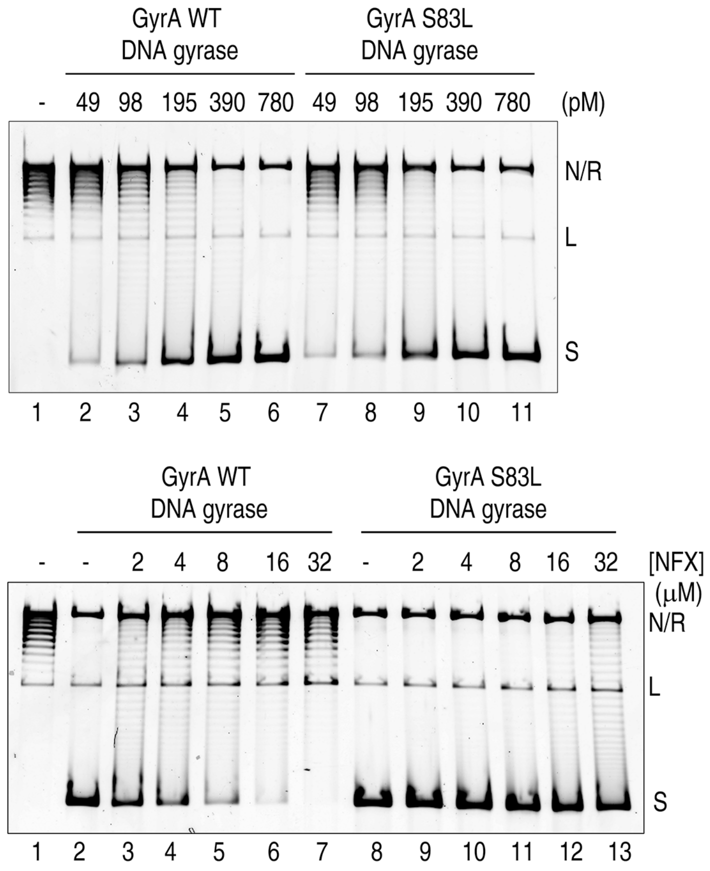

Supplementary Figure 5. GyrA S83L DNA gyrase is as active as wild-type DNA gyrase. Top panel, the indicated concentrations of GyrA S83L and wild-type gyrase were assayed in a DNA supercoiling assay using relaxed pCG09 DNA as described under Materials and Methods. Bottom panel, 780 pM wild-type and GyrA S83L gyrase were incubated with either no or the indicated concentrations of norfloxacin in DNA supercoiling assays using relaxed pCG09 DNA as described under Materials and Methods. N/R, nicked, relaxed; L, linear; S, supercoiled.

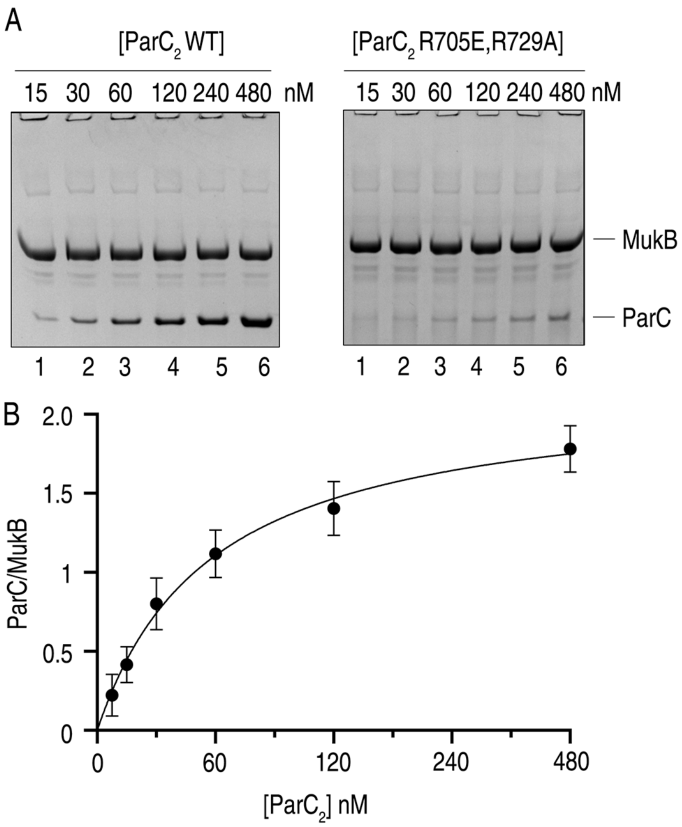

Supplementary Figure 6. Binding of ParC to MukB. MukB was immobilized on protein A-sepharose beads using affinity-purified polyclonal rabbit ant-MukB antibodies. Binding reactions containing 125 nM MukB and the indicated concentrations of either wild-type or R703E R729A ParC were incubated for 15 min at 1250 rpm and 37 °C in a thermomixer. Unbound ParC was removed, the beads washed, and the proteins released from the beads resolved on SDS polyacrylamide as described under Materials and Methods. Shown are the mean and standard deviation (n=4).
